#  Molecular Characterization of Superficial Layers of the Presubiculum During Development

**DOI:** 10.3389/fnana.2021.662724

**Published:** 2021-05-24

**Authors:** Jiayan Liu, Tetsuhiko Kashima, Shota Morikawa, Asako Noguchi, Yuji Ikegaya, Nobuyoshi Matsumoto

**Affiliations:** ^1^Graduate School of Pharmaceutical Sciences, The University of Tokyo, Tokyo, Japan; ^2^Institute for AI and Beyond, The University of Tokyo, Tokyo, Japan; ^3^Center for Information and Neural Networks (CiNet), National Institute of Information and Communications Technology, Suita City, Japan

**Keywords:** presubiculum, anterior thalamus, vesicular glutamate transporter 2, adeno-associated virus, mouse, calbindin, interneuron, development

## Abstract

The presubiculum, a subarea of the parahippocampal region, plays a critical role in spatial navigation and spatial representation. An outstanding aspect of presubicular spatial codes is head-direction selectivity of the firing of excitatory neurons, called head-direction cells. Head-direction selectivity emerges before eye-opening in rodents and is maintained in adulthood through neurophysiological interactions between excitatory and inhibitory neurons. Although the presubiculum has been physiologically profiled in terms of spatial representation during development, the histological characteristics of the developing presubiculum are poorly understood. We found that the expression of vesicular glutamate transporter 2 (VGluT2) could be used to delimit the superficial layers of the presubiculum, which was identified using an anterograde tracer injected into the anterior thalamic nucleus (ATN). Thus, we immunostained slices from mice ranging in age from neonates to adults using an antibody against VGluT2 to evaluate the VGluT2-positive area, which was identified as the superficial layers of the presubiculum, during development. We also immunostained the slices using antibodies against parvalbumin (PV) and somatostatin (SOM) and found that in the presubicular superficial layers, PV-positive neurons progressively increased in number during development, whereas SOM-positive neurons exhibited no increasing trend. In addition, we observed repeating patch structures in presubicular layer III from postnatal days 12. The abundant expression of VGluT2 suggests that the presubicular superficial layers are regulated primarily by VGluT2-mediated excitatory neurotransmission. Moreover, developmental changes in the densities of PV- and SOM-positive interneurons and the emergence of the VGluT2-positive patch structures during adolescence may be associated with the functional development of spatial codes in the superficial layers of the presubiculum.

## Introduction

Within the parahippocampal region surrounding the hippocampus (Furtak et al., 2007; van Strien et al., 2009; Olsen et al., 2017), the presubiculum is located between the hippocampus and entorhinal cortex (van Strien et al., 2009) and innervated by the subcortical areas (van Groen and Wyss, 1990a). The presubiculum plays a pivotal role in spatial navigation (Taube et al., 1992) and spatial representation (Taube et al., 1990a, b; Taube, 2007; Boccara et al., 2010; Yoder et al., 2011; Tukker et al., 2015). For spatial representation, presubicular excitatory neurons increase their firing rates exclusively when an animal’s head points in a specific direction. These presubicular neurons are called head-direction cells (Taube et al., 1990a, b; Taube, 2007; Yoder et al., 2011; Tukker et al., 2015). The presubiculum is divided into six layers (van Groen and Wyss, 1990b), and head-direction cells are found primarily in the superficial layers (especially layer III; Preston-Ferrer et al., 2016). Extensive studies have identified the neural circuits responsible for the generation of head-direction signals (Taube, 2007). Directional signals originate from integrated vestibular information (Stackman and Taube, 1997) and are updated by motor and proprioceptive feedback systems (Taube and Burton, 1995). Such idiothetic (i.e., internal to the body) information is undoubtedly available before eye-opening, thus leading to the emergence of coherent head-direction signals in rodents prior to weaning (Bjerknes et al., 2015). Moreover, an activity-dependent inhibitory feedback loop *via* somatostatin (SOM)-expressing (not parvalbumin (PV)-expressing) interneurons may underlie self-sustained head-direction signals in superficial layers of the presubiculum of adult mice (Simonnet et al., 2017). The physiological properties of the presubiculum have undergone experimental and theoretical investigation with a focus on spatial representation; however, the histological features of the presubicular superficial layers during development remain largely undetermined.

To address this issue, we first defined the presubicular superficial layers by injection of an anterograde tracer into the anterior thalamic nucleus (ATN; Robertson and Kaitz, 1981; Shibata, 1993; Nassar et al., 2018; Mathiasen et al., 2020). We then attempted to delimit superficial layers of the presubiculum based on vesicular glutamate transporter 2 (VGluT2) expression because VGluT2 has been previously reported to be abundantly expressed in a downstream region of the subiculum (Wouterlood et al., 2008; Ishihara and Fukuda, 2016; Kashima et al., 2019). Hence, we immunostained slices prepared from tracer-injected mice using an antibody against VGluT2 to confirm the overlap of the two signals. We then took advantage of VGluT2 immunostaining to delimit the superficial layers of the presubiculum in neonatal to adult mice and analyzed the distribution of VGluT2. We finally immunostained slices using antibodies against PV and SOM and quantified presubicular PV- and SOM-expressing interneurons based on their immunosignals during development to histologically characterize the superficial layers of the presubiculum.

## Materials and Methods

### Animal Ethics

Animal experiments were performed with the approval of the Animal Experiment Ethics Committee of the University of Tokyo (approval number: P29–15) and in accordance with the University of Tokyo guidelines for the care and use of laboratory animals. The experimental protocols were performed in accordance with the Fundamental Guidelines for the Proper Conduct of Animal Experiments and Related Activities in Academic Research Institutions (Ministry of Education, Culture, Sports, Science and Technology, Notice No. 71 of 2006), the Standards for Breeding and Housing of and Pain Alleviation for Experimental Animals (Ministry of the Environment, Notice No. 88 of 2006) and the Guidelines on the Method of Animal Disposal (Prime Minister’s Office, Notice No. 40 of 1995).

A total of 40 animals were housed in groups (unless otherwise specified) under conditions of controlled temperature and humidity (22 ± 1°C, 55 ± 5%) and maintained on a 12:12-h light/dark cycle (lights on from 7:00 to 19:00) with *ad libitum* access to food and water unless otherwise specified. All efforts were made to minimize animal suffering.

### Adeno-Associated Virus Production

Adeno-associated virus (AAV) vectors were generated as previously described (Miyawaki et al., 2020). pAAV-hSyn-Synaptophysin-mCherry-WPRE-hGH-polyA was constructed from pAAV-hSyn-GFP-WPRE-hGH-polyA (Addgene plasmid #50465), synaptophysin (Exons 2–6) and mCherry using KpnI-HF (R3142S, New England Biolabs Japan, Tokyo, Japan) and HindIII-HF (R3104S, New England Biolabs Japan). We cloned mCherry into the vector for expression as a fusion to the C-terminal region of synaptophysin using the In-Fusion HD Cloning Kit (Z9648N, Takara Bio Inc., Shiga, Japan). Recombinant AAV was generated by triple transfection of the 293 AAV cell line (AAV-100, Cell Biolabs, Inc., CA, USA) with AAVdj rep-cap and pHelper from the AAV-DJ Helper Free Packaging System (VPK-400-DJ, Cell Biolabs, Inc.) and pAAV-hSyn-Synaptophysin-mCherry-WPRE-hGH-polyA using PEI-max (24765, Polysciences, Inc., PA, USA). AAV vectors were purified using the AAVpro Purification Kit All Serotypes (6666, Takara Bio Inc.). Virus titers were then determined by qPCR using the primer pair AAV2 ITR (Aurnhammer et al., 2012), Luna Universal® qPCR Master Mix (M3003S, New England Biolabs, MA, USA), and the LightCycler® qPCR 2.0 system (DX400, Roche, Basel, Switzerland).

### Stereotaxic Injection

**Figure 1:**
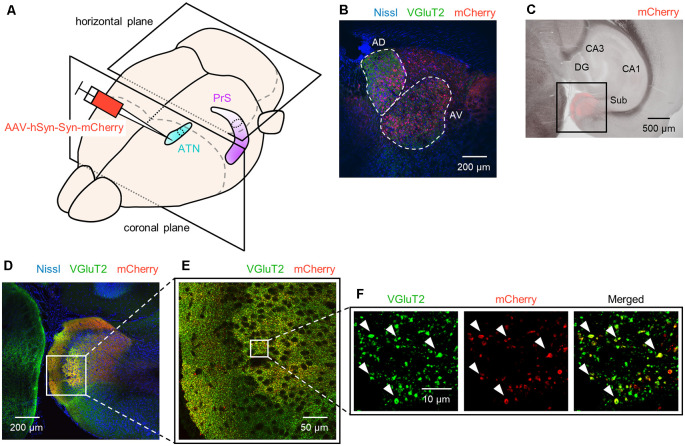
Anterograde tracing of the presubiculum by injection of adeno-associated viral (AAV) vectors into the ATN. **(A)** Experimental diagram. AAV (red) was injected into the ATN (*light blue*). Two-week after the injection, the PrS (*purple*) was anterogradely traced. After perfusion and postfixation, the brain was first coronally sectioned from the rostral end to confirm the injection site (i.e., ATN), followed by horizontal sectioning from the dorsal end for the presubiculum. **(B)** Representative immunohistochemical image (10×) of the injection site. Nissl, anti-vesicular glutamate transporter 2 (VGluT2), and mCherry signals are represented by *blue*, *green*, and *red*, respectively. Note that in the current study, the ATN is subdivided into the anterior dorsal thalamus (AD) and the anterior ventral thalamus (AV), each of which is encircled by *white* dashed lines. **(C)** Low-magnification image (4×) of the hippocampus and parahippocampal region in a slice of an AAV-injected mouse. The AAV-mediated anterograde tracing signal was confined to a region downstream of the subiculum. **(D)** Fluorescent image (10×) of the boxed area in **(C)**. **(E)** Magnified image (40×) of the boxed area in **(D)**. **(F)** Magnified image (60× objective and 2× electronic zoom) of the boxed area in **(E)**. Note that synaptic terminals or boutons from ATN (*red*) include VGluT2 (*green*), suggesting that ATN axons target presubicular neurons. ATN, anterior thalamic nucleus; PrS, presubiculum; DG, dentate gyrus; Sub, subiculum.

Six 29-to 35-day-old male C57BL/6J mice (Japan SLC, Shizuoka, Japan) with preoperative weights of approximately 20 g underwent stereotaxic surgery. Briefly, general anesthesia for mice was induced and maintained by inhalation of 4% (v/v, in air) and 1–2% isoflurane gas, respectively, with careful inspection of the animal’s condition during the entire surgical procedure. The veterinary ointment was applied to the eyes of the mouse to prevent drying. The skin was sterilized with povidone-iodine and 70% ethanol prior to incision. After anesthesia, we mounted the mouse onto the stereotaxic apparatus with ear bars and a nose clamp. The scalp was removed with surgical scissors, and circular craniotomy with a diameter of approximately 0.9 mm was performed using a high-speed dental drill. We injected AAVdj-hSyn-synaptophysin-mCherry into the ATN (0.82 mm posterior and 0.80 mm lateral to Bregma and 2.6 mm from the pia mater) at a speed of 100 nl/min to deliver 200 nl using a Hamilton syringe and a syringe pump controller. The viral titer was between 1.60 × 10^13^ GC/ml and 3.19 × 10^13^ GC/ml. Note that the ATN is often subdivided into the anterodorsal thalamic nucleus (AD), the anteroventral thalamic nucleus (AV), and the anteromedial thalamic nucleus (Jankowski et al., 2013; Franklin and Paxinos, 2019), but we refer to the AD and AV as the ATN in this study (Figure 1F) in accordance with a previous investigation (Nassar et al., 2018).

Following surgery, each mouse was allowed to recover from anesthesia and housed individually in a cage with free access to water and food for 2 week; note that anterograde labeling was sufficiently expressed at 2 week after injection.

### Histology

Intact postnatal (P) 5-, 6-, 7-, 8-, 10-, 12-, 15-, and 21-day-old, 6-week-old, and postsurgery (i.e., 43-to 49-day-old) C57BL/6J mice were used for immunostaining (*n* = 6 mice for postsurgery, 3 mice at P5 day old and 6 week old, and 4 mice at other ages; that is, *n* = 40 mice in total). P5 and P6 mice were anesthetized on ice, while the mice of other ages were anesthetized *via* intraperitoneal administration of 150 mg/ml urethane dissolved in saline. After general anesthesia was confirmed by the lack of reflex responses to tail and toe pinch, the mice were transcardially perfused with ice-cold 0.01 M phosphate-buffered saline (PBS) followed by 4% paraformaldehyde in PBS and decapitated. The brains were carefully removed and postfixed in 4% paraformaldehyde in PBS overnight. The brains of AAV-injected mice were first coronally sectioned from the anterior region to confirm the injection site (i.e., ATN) and then horizontally sectioned from the dorsal region (Figure 1A). The brains of all other mice were horizontally sectioned throughout. When horizontally sectioning the brain, we glued the connection between the cerebrum and the pons onto the tray (Supplementary Figure 1). The brain was glued so that the brain surface would be as parallel to the tray as possible. All brains were sectioned using a vibratome at a thickness of 100 μm. A given section of each brain was defined as 0 μm (i.e., the most dorsal) when the cross-sectional area of the hippocampal pyramidal cell layer (*stratum pyramidale*) exceeded 0.3 mm^2^. The coordinate of this zero-reference along the dorsal-to-ventral axis was approximately 1,400 μm beneath the brain surface (Supplementary Figure 1). For subsequent immunohistochemistry, we took sections at intervals of 300 μm.

All sections were blocked with 5% bovine serum albumin (BSA) and 0.3% Triton X-100 in PBS for 1 h at room temperature.

(I) For the AAV-injected mice, the sections were incubated with the appropriate combination of the following primary antibodies: (i) guinea pig primary antibody against vesicular glutamate transporter 2 (VGluT2, also known as DNPI (Aihara et al., 2002); 1:500, VGluT2-GP-Af810, Frontier Institute, Hokkaido, Japan); (ii) mouse primary antibody against calbindin (1:200, 214211, Synaptic Systems, Goettingen, Germany); (iii) rabbit primary antibody against PSD95 (1:500, PSD95-Rb-Af1720, Frontier Institute); (iv) mouse primary antibody against MAP2 (1:500, MAB378, Merck, Darmstadt, Germany); and (v) mouse primary antibody against VGluT1 (1:500, 135311, Synaptic Systems) in 5% BSA and 0.3% Triton X-100 in PBS for 16 h at room temperature. The sections were washed with PBS for 10 min three times and then incubated with NeuroTrace 435/455 blue fluorescent Nissl stain (1:200, N21479, Thermo Fisher Scientific, MA, USA) and the appropriate combination of the following secondary antibodies: (i) Alexa Fluor 488-conjugated goat secondary antibody against guinea pig IgG (1:500, A11073, Thermo Fisher Scientific); (ii) Alexa Fluor 647-conjugated goat secondary antibody against mouse IgG (1:200, A21236, Thermo Fisher Scientific); (iii) Alexa Fluor 647-conjugated goat secondary antibody against rabbit IgG (1:500, A21245, Thermo Fisher Scientific); and (iv) Alexa Fluor 405-conjugated goat secondary antibody against mouse IgG (1:500, A31553, Thermo Fisher Scientific) in 5% BSA and 0.3% Triton X-100 in PBS for 5 h at room temperature. Note that we did not perform Nissl staining in the experiments for Supplementary Figures 13, 14.
(II) For other mice, the sections were incubated with: (i) guinea pig primary antibody against VGluT2 (1:500), rabbit primary antibody against PV (1:500, PV-Rb-Af750, Frontier Institute), and mouse primary antibody against SOM (1:200, SOM-018, GeneTex, CA, USA); or (ii) guinea pig primary antibody against VGluT2 (1:500), mouse primary antibody against SOM (1:200), and rabbit primary antibody against GABA (1:500, A2052, Merck) in 5% BSA and 0.3% Triton X-100 in PBS for 48 h at 4°C. The sections were washed with PBS for 10 min three times and then incubated with NeuroTrace 435/455 blue fluorescent Nissl stain (1:200), and (i) Alexa Fluor 488-conjugated goat secondary antibody against guinea pig IgG (1:500), Alexa Fluor 594-conjugated goat secondary antibody against rabbit IgG (1:500, A11037, Thermo Fisher Scientific), and Alexa Fluor 647-conjugated goat secondary antibody against mouse IgG (1:200) or (ii) Alexa Fluor 488-conjugated goat secondary antibody against guinea pig IgG (1:500), Alexa Fluor 594-conjugated goat secondary antibody against mouse IgG (1:500, A11032, Thermo Fisher Scientific), and Alexa Fluor 647-conjugated goat secondary antibody against rabbit IgG (1:500, A21245, Thermo Fisher Scientific) in 5% BSA and 0.3% Triton X-100 in PBS for 24 h at 4°C.

### Image Acquisition

For all figures other than Figure 1C and Supplementary Figures 1B,C the images (1,024 × 1,024 pixels, 16-bit intensity) for each region of interest (ROI) were acquired at *Z*-intervals of 2.0 μm (10×) or 0.5 μm (20×, 40×, and 60×) using a confocal microscope (FV1000, Olympus, Tokyo, Japan) equipped with 10×, 20×, 40×, and 60× objective lens and Z-stacked using ImageJ software (National Institutes of Health, Bethesda, MD, USA). For Figure 1C and Supplementary Figures 1B,C images were acquired using a phase-contrast microscope (BZ-X710, Keyence, Osaka, Japan) equipped with 4× objectives. We used optical zoom by objective lens (described above) but further magnified some images with digital (i.e., electronic) zoom if necessary (Figure 1F, Supplementary Figures 3, 13, 14B).

### Data Analysis

The data were analyzed using MATLAB (MathWorks, MA, USA). The summarized data are displayed as box-and-whisker plots. Representative values are reported as the mean ± standard error of the mean (SEM) unless otherwise specified. *P* < 0.05 was considered statistically significant.

Each image was split into RGB mode using ImageJ software. We then obtained the color intensity of each pixel of the three-color images to calculate the spatial correlation (Figure 3). We created three matrices that included such elements as the pixel ID numbers and the color intensities and calculated Pearson’s correlations between two of the three matrices (Noguchi et al., 2017). We determined the areas of the superficial layers of the presubiculum by outlining the VGluT2-positive areas and calculated the area using ImageJ software. The cell densities of SOM-expressing and PV-expressing interneurons were calculated by dividing the number of immunopositive cells by the area of the superficial layers of the presubiculum.

**Figure 2:**
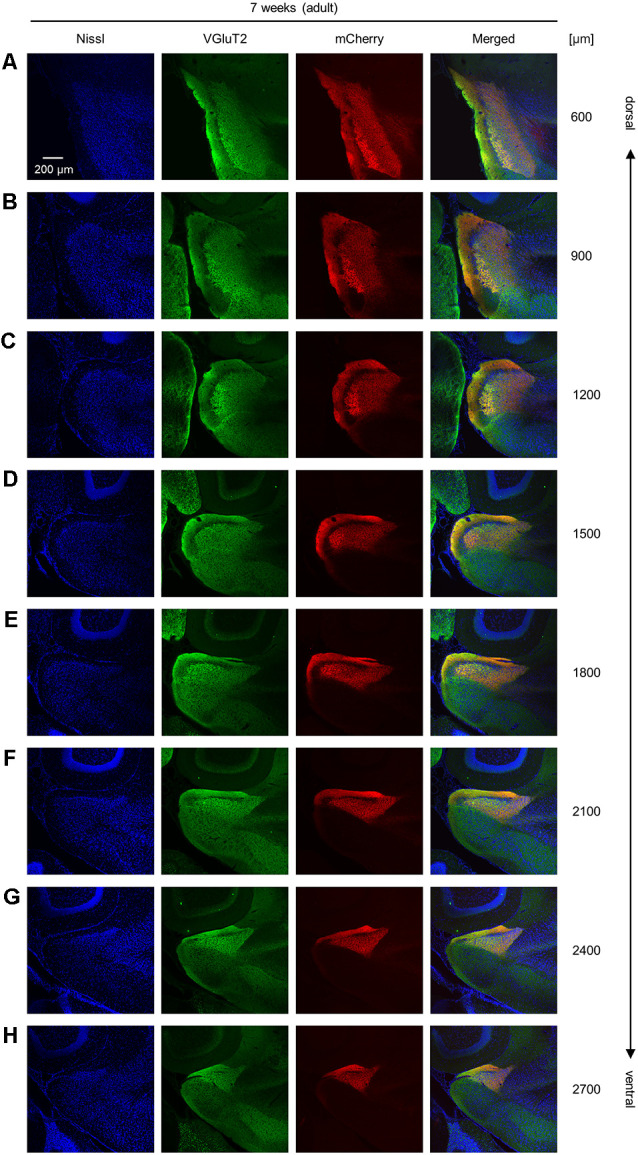
Representative photographs of VGluT2 immunoreactivity and AAV-mediated anterograde tracing in superficial layers of the presubiculum from an adult mouse along the dorsoventral axis. **(A)** Superficial layers of the presubiculum were stained for Nissl substances (*blue*, *leftmost* (*first*)) and VGluT2 (*green*, *second*) and simultaneously visualized with AAV-mediated anterograde tracing (*red*, *third*) in a slice at 600 μm depth. A merged image is displayed in the *fourth* panel. The dorsoventral level was indicated as the distance (μm) from the most dorsal section (i.e., 0 μm). **(B–H)** The same as **(A)** but at 900 μm, 1,200 μm, 1,500 μm, 1,800 μm, 2,100 μm, 2,400 μm, and 2,700 μm, respectively. VGluT2, vesicular glutamate transporter 2; AAV, adeno-associated virus.

**Figure 3:**
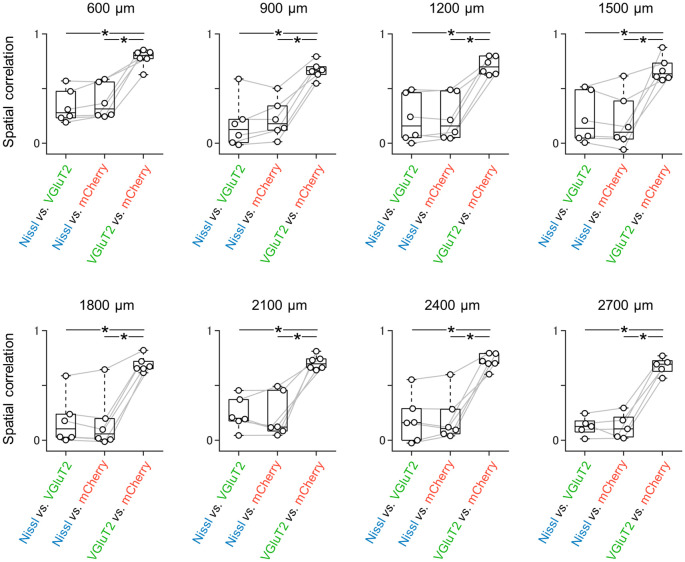
Spatial correlations between all possible pairs of three signals at different dorsoventral levels. Spatial correlations were calculated between all possible pairs of signals (i.e., Nissl *vs*. VGluT2, Nissl vs. mCherry (as a result of AAV-mediated anterograde tracing), and VGluT2 vs. mCherry). At either dorsoventral level, where the most dorsal section is defined as 0 μm, the spatial correlations between VGluT2 and mCherry were significantly higher than those between the other two pairs (*n* = 5–6 mice, *P < 0.05, paired *t*-test with *post hoc* Bonferroni correction). VGluT2, vesicular glutamate transporter 2; AAV, adeno-associated virus.

## Results

### VGluT2 Expression-Based Identification of the Superficial Layers of the Mouse Presubiculum During Postnatal Development

We unilaterally injected AAV into the ATN of adult mice to visualize axon terminals in the presubiculum on the basis of mCherry expression. After the 2-week postinjection period, we prepared 100-μm-thick coronal sections from the anterior side, examined the thalamic injection site, and then horizontally sectioned the remaining brain from the dorsal end at a thickness of 100 μm (Figure 1, Supplementary Figure 2). Consistent with a previous study (Nassar et al., 2018), anterograde tracing by AAV confirmed that thalamic (i.e., ATN) axonal innervation was restricted in the extrahippocampal region, which was defined as the superficial layers (i.e., layers I, II, and III) of the presubiculum (Figure 2, Supplementary Figure 2). Since we and others have previously found that VGluT2 immunoreactivity was also confined downstream of the subiculum (Wouterlood et al., 2008; Ishihara and Fukuda, 2016; Kashima et al., 2019), we immunostained VGluT2 in brain slices prepared from AAV-injected mice and counterstained them with blue Nissl stains (Figure 2). We calculated the spatial correlation between two of the three fluorescence channels (i.e., Nissl *vs*. VGluT2, Nissl *vs*. mCherry, and VGluT2 *vs*. mCherry) to quantify the overlap between the VGluT2-immunopositive area and the presubicular superficial layers defined by thalamic projection. Spatial correlations between VGluT2 and mCherry (thalamic innervation) were significantly higher than those between the other two pairs (i.e., Nissl *vs*. VGluT2, Nissl *vs*. mCherry) at all dorsoventral (i.e., dorsal-to-ventral, hereafter) levels (Figure 1F, Supplementary Tables 1, 2). The high spatial correlations between VGluT2 and mCherry signals are almost independent of the magnification of objectives [0.71 ± 0.03 (20×), 0.60 ± 0.04 (60×), *n* = 3 mice each; see also Supplementary Figure 3, Supplementary Table 1]. In the dorsal portion of the presubiculum (namely, 600 μm, 900 μm, 1,200 μm from the dorsomost section (Figure 2), the overlap of VGluT2 and mCherry signals was stronger in the distal region than in the proximal region mainly due to the repeating patch structures (precisely described below; Figure 14). On the other hand, the overlap of the two signals was homogeneous in the intermediate and ventral portions of the presubiculum (i.e., 1,500 μm, 1,800 μm, 2,100 μm, 2,400 μm, 2,700 μm). The proportion of mCherry signals that contained VGluT2 immunosignals was higher than 90% based on high–magnification images (Supplementary Figure 3). The high correlation of the overlap between the two signals (in the high–magnification images) was consistent with the spatial correlation (Figure 3). Moreover, the distribution of VGluT2 immunosignals delineated the presubicular superficial layer and distinguished the presubiculum from the surrounding areas, including the hippocampus, the subiculum, the parasubiculum, and the entorhinal cortex (Supplementary Figure 1). Therefore, we identified the superficial layers of the presubiculum as the region delimited by VGluT2 expression for subsequent analyses. Note that the boundary line between the presubiculum and the parasubiculum was more ambiguous than that of the presubiculum and the subiculum for adult mice. Compared with adults, we could not necessarily exclude the possibility that the manually drawn boundary line between the presubiculum and the parasubiculum stepped into the “true” parasubiclum for neonatal and juvenile mice.

**Figure 4:**
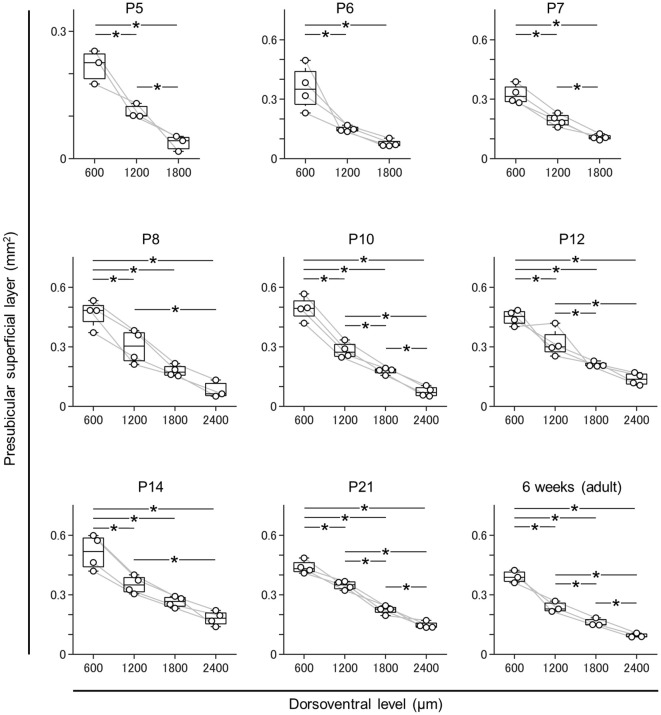
Dorsal-to-ventral decrease in the area of the superficial layers of the presubiculum across all ages. The superficial layers of the presubiculum were determined based on VGluT2 immunofluorescence. The area of the superficial layers was estimated at 600 μm, 1,200 μm, 1,800 μm, and 2,400 μm (at ages older than P7) across all ages tested (i.e., P5, P6, P7, P8, P10, P12, P14, P21, and 6 week old). At all ages, the area tended to decrease from the dorsal to ventral end (*n* = 3–4 mice, *P < 0.05, Tukey–Kramer test).

**Figure 5:**
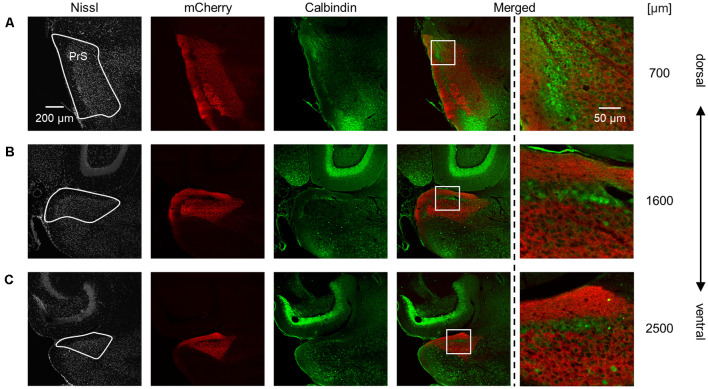
Complementary pattern of AAV-mediated tracing and calbindin immunosignals in the superficial layers of the presubiculum. **(A)** Presubiculum (at 700 μm from the most dorsal section) was imaged by staining Nissl substances (*gray*, *leftmost* (*first*)), AAV-mediated fluorescent tracing (*red*, *second*), and immunostaining of calbindin (*green*, *third*). The merged signal (except for Nissl) is shown in the *fourth* panel. Calbindin is enriched in layer II in the presubiculum, whereas mCherry signals are evident in layers I and III. The presubicular superficial layers are indicated by a *white* loop in the *first* panel. A magnified image of the *white* boxed area in the *fourth* panel is displayed in the *fifth* panel. **(B,C)** The same as **(A)** but at 1,600 μm and 2,500 μm, respectively. PrS, presubiculum; AAV, adeno-associated virus.

**Figure 6:**
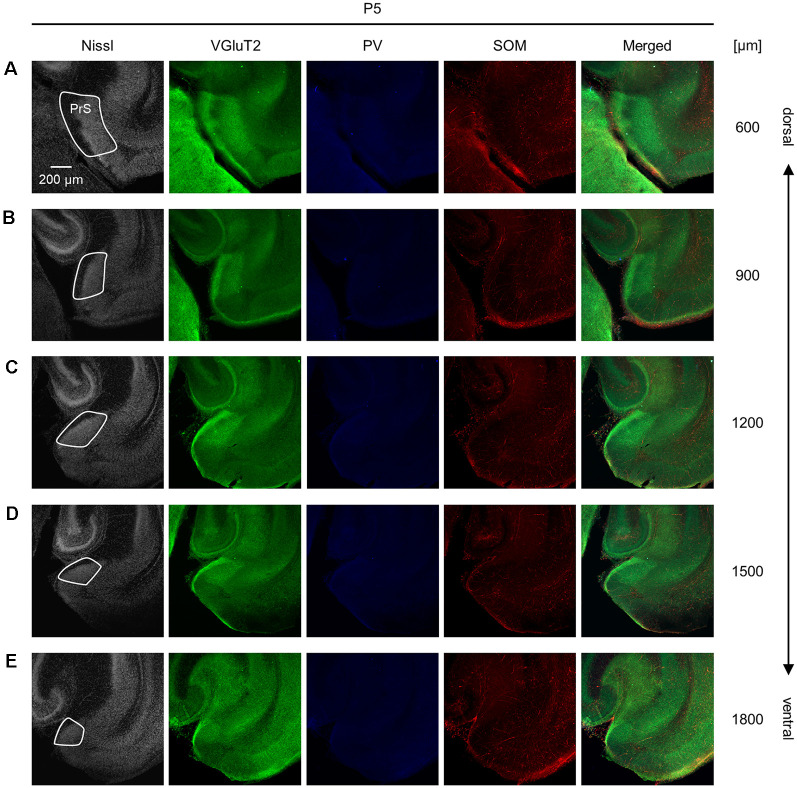
Representative photographs of the presubicular superficial layers of a postnatal 5-day-old mouse.*** (*A)** Superficial layers of the presubiculum of a postnatal 5-day-old mouse were stained for Nissl substances (*gray*, *leftmost* (*first*)) and immunostained for VGluT2 (*green*, *second*), parvalbumin (PV; *blue*, *third*), and somatostatin (SOM; *red*, *fourth*) in a slice at 600 μm depth. A merged image (except for Nissl) is displayed in the *fifth* panel. The presubicular superficial layers are delineated by a *white* loop in the *first* panel. The dorsoventral level is indicated as the distance (μm) from the most dorsal section (i.e., 0 μm). **(B–E)** The same as **(A)** but at 900 μm, 1,200 μm, 1,500 μm, and 1,800 μm, respectively. PrS, presubiculum; VGluT2, vesicular glutamate transporter 2; PV, parvalbumin; SOM, somatostatin.

**Figure 7:**
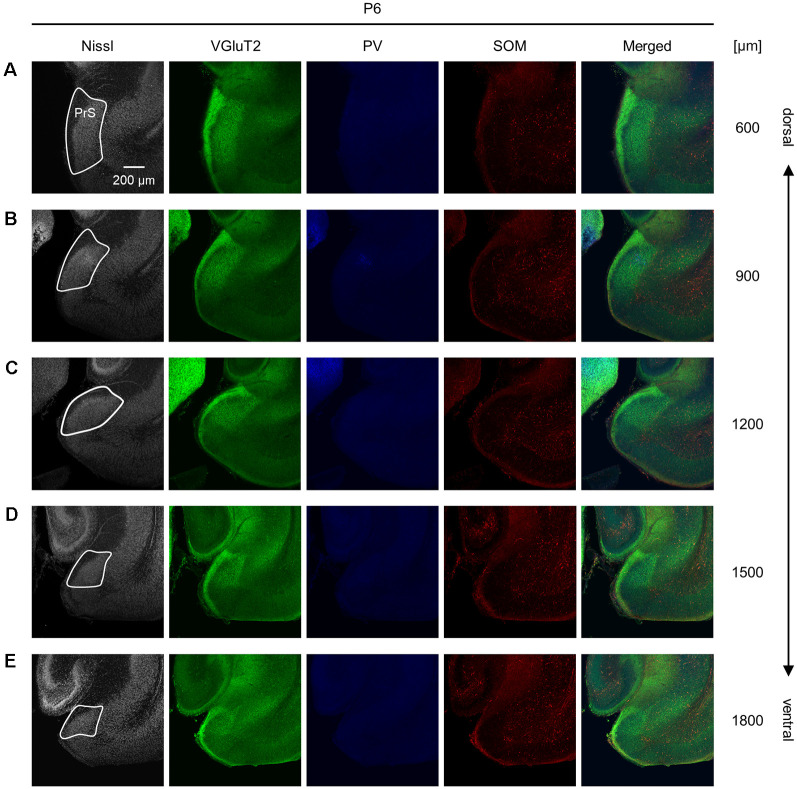
Representative photographs of the presubicular superficial layers of a postnatal 6-day-old mouse. **(A)** Superficial layers of the presubiculum of a postnatal 6-day-old mouse were stained for Nissl substances (*gray*, *leftmost* (*first*)) and immunostained for VGluT2 (*green*, *second*), parvalbumin (PV; *blue*, *third*), and somatostatin (SOM; *red*, *fourth*) in a slice at 600 μm depth. A merged image (except for Nissl) is displayed in the *fifth* panel. The presubicular superficial layers are delineated by a *white* loop in the *first* panel. The dorsoventral level is indicated as the distance (μm) from the most dorsal section (i.e., 0 μm).**(B–E)** The same as **(A)** but at 900 μm, 1,200 μm, 1,500 μm, and 1,800 μm, respectively. PrS, presubiculum; VGluT2, vesicular glutamate transporter 2; PV, parvalbumin; SOM, somatostatin.

**Figure 8:**
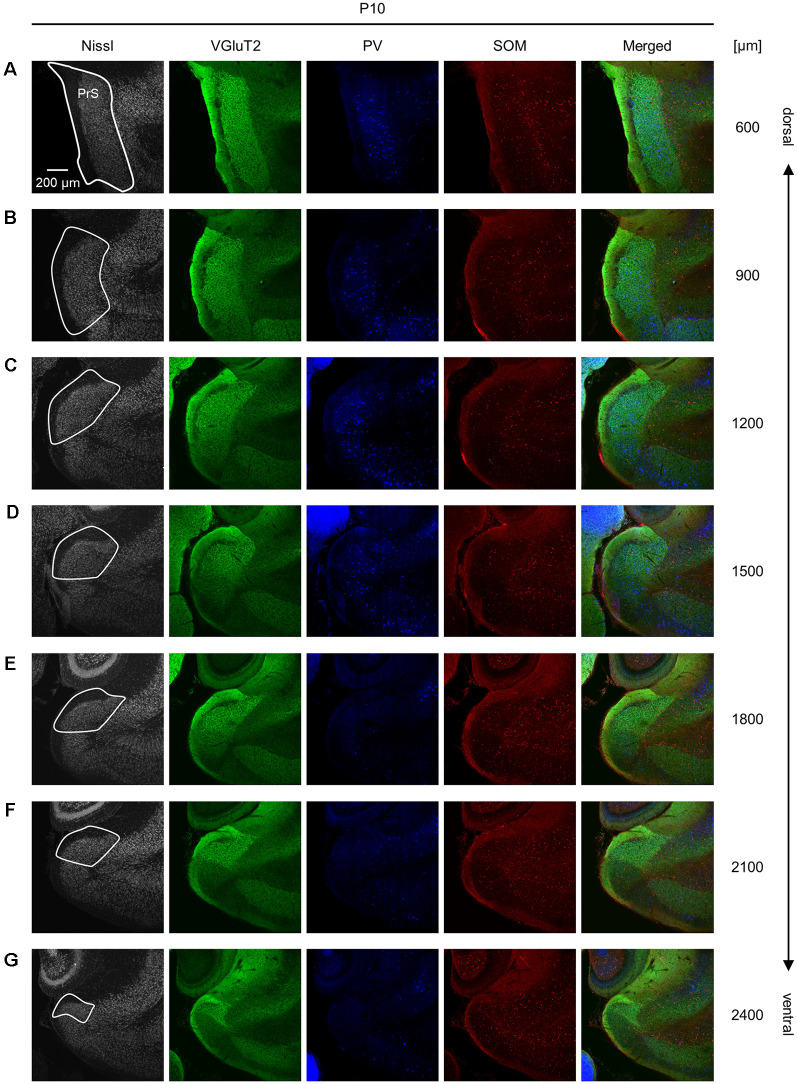
Representative photographs of the presubicular superficial layers of a postnatal 10-day-old mouse. **(A)** Superficial layers of the presubiculum of a postnatal 10-day-old mouse were stained for Nissl substances (*gray*, *leftmost* (*first*)) and immunostained for VGluT2 (*green*, *second*), parvalbumin (PV; *blue*, *third*), and somatostatin (SOM; *red*, *fourth*) in a slice at 600 μm depth. A merged image (except for Nissl) is displayed in the *fifth* panel. The presubicular superficial layers are delineated by a *white* loop in the *first* panel. The dorsoventral level is indicated as the distance (μm) from the most dorsal section (i.e., 0 μm). **(B–G)** The same as **(A)**, but at 900 μm, 1,200 μm, 1,500 μm, 1,800 μm, 2,100 μm, and 2,400 μm, respectively. PrS, presubiculum; VGluT2, vesicular glutamate transporter 2; PV, parvalbumin; SOM, somatostatin.

**Figure 9:**
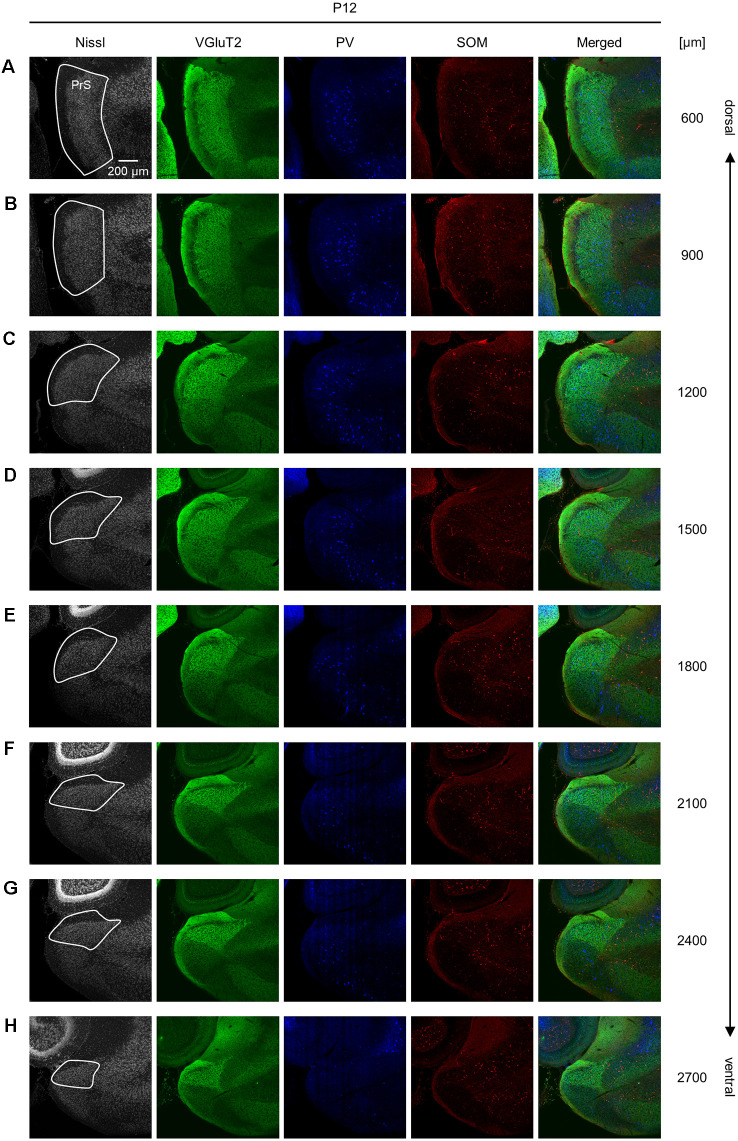
Representative photographs of the presubicular superficial layers of a postnatal 12-day-old mouse. **(A)** Superficial layers of the presubiculum of a postnatal 12-day-old mouse were stained for Nissl substances (*gray*, *leftmost* (*first*)) and immunostained for VGluT2 (*green*, *second*), parvalbumin (PV; *blue*, *third*), and somatostatin (SOM; *red*, *fourth*) in a slice at 600 μm depth. A merged image (except for Nissl) is displayed in the *fifth* panel. The presubicular superficial layers are delineated by a *white* loop in the *first* panel. The dorsoventral level is indicated as the distance (μm) from the most dorsal section (i.e., 0 μm). **(B–H)** The same as **(A)** but at 900 μm, 1,200 μm, 1,500 μm, 1,800 μm, 2,100 μm, 2,400 μm, and 2,700 μm, respectively. PrS, presubiculum; VGluT2, vesicular glutamate transporter 2; PV, parvalbumin; SOM, somatostatin.

**Figure 10:**
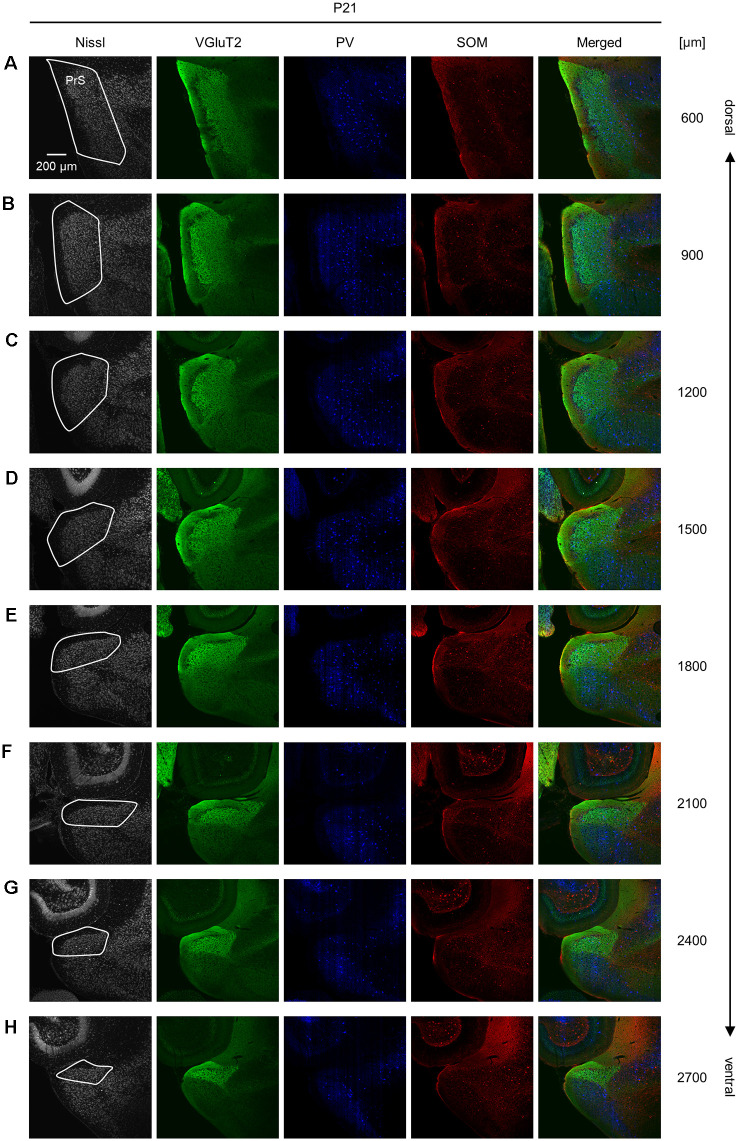
Representative photographs of the presubicular superficial layers of a postnatal 21-day-old mouse. **(A)** Superficial layers of the presubiculum of a postnatal 21-day-old mouse were stained for Nissl substances (*gray*, *leftmost* (*first*)) and immunostained for VGluT2 (*green*, *second*), parvalbumin (PV; *blue*, *third*), and somatostatin (SOM; *red*, *fourth*) in a slice at 600 μm depth. A merged image (except for Nissl) is displayed in the *fifth* panel. The presubicular superficial layers are delineated by a *white* loop in the *first* panel. The dorsoventral level is indicated as the distance (μm) from the most dorsal section (i.e., 0 μm). **(B–H)** The same as **(A)** but at 900 μm, 1,200 μm, 1,500 μm, 1,800 μm, 2,100 μm, 2,400 μm, and 2,700 μm, respectively. PrS, presubiculum; VGluT2, vesicular glutamate transporter 2; PV, parvalbumin; SOM, somatostatin.

**Figure 11:**
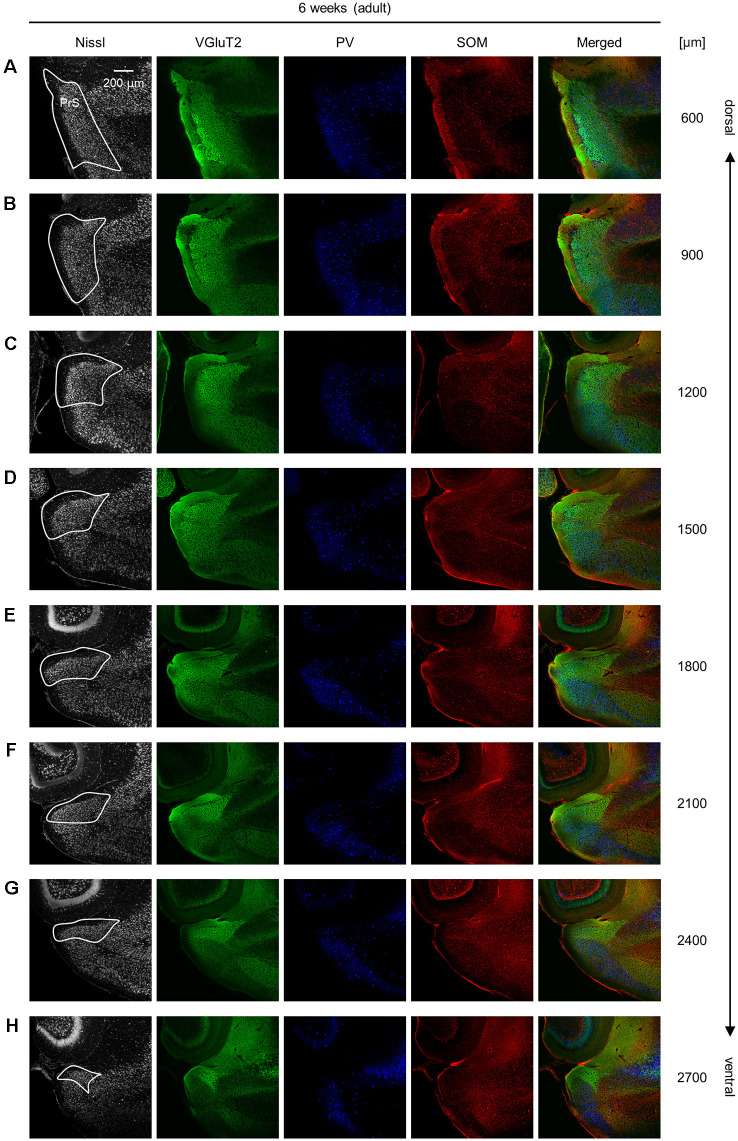
Representative photographs of the presubicular superficial layers of a 6-week-old mouse. **(A)** Superficial layers of the presubiculum of a 6-week-old (adult) mouse were stained for Nissl substances (*gray*, *leftmost* (*first*)) and immunostained for VGluT2 (*green*, *second*), parvalbumin (PV; *blue*, *third*), and somatostatin (SOM; *red*, *fourth*) in a slice at 600 μm depth. A merged image (except for Nissl) is displayed in the *fifth* panel. The presubicular superficial layers are delineated by a *white* loop in the *first* panel. The dorsoventral level is indicated as the distance (μm) from the most dorsal section (i.e., 0 μm). **(B–H)** The same as **(A)** but at 900 μm, 1,200 μm, 1,500 μm, 1,800 μm, 2,100 μm, 2,400 μm, and 2,700 μm, respectively. PrS, presubiculum; VGluT2, vesicular glutamate transporter 2; PV, parvalbumin; SOM, somatostatin.

**Figure 12:**
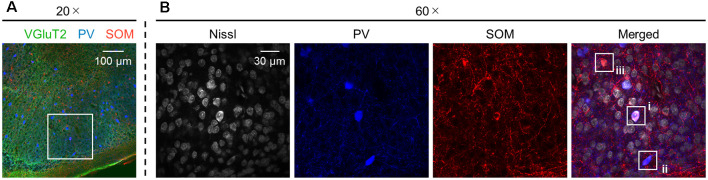
Presubicular neurons coexpressing PV and SOM. **(A)** Representative image (20×) of the superficial layers of the adult (6-week-old) presubiculum immunostained for VGluT2 (*green*), PV (*blue*), and SOM (*red*). **(B)** High-magnification (60×) image of the *white* boxed area in **(A)**. The section was stained for Nissl substances (*gray*, *leftmost* (*first*)) and immunostained for PV (*blue*, *second*) and SOM (*red*, *third*). The merged image is displayed in the *fourth* panel, which shows (i) neurons coexpressing both PV and SOM and (ii and iii) neurons expressing PV alone and SOM alone, respectively. VGluT2, vesicular glutamate transporter 2; PV, parvalbumin; SOM, somatostatin.

**Figure 13:**
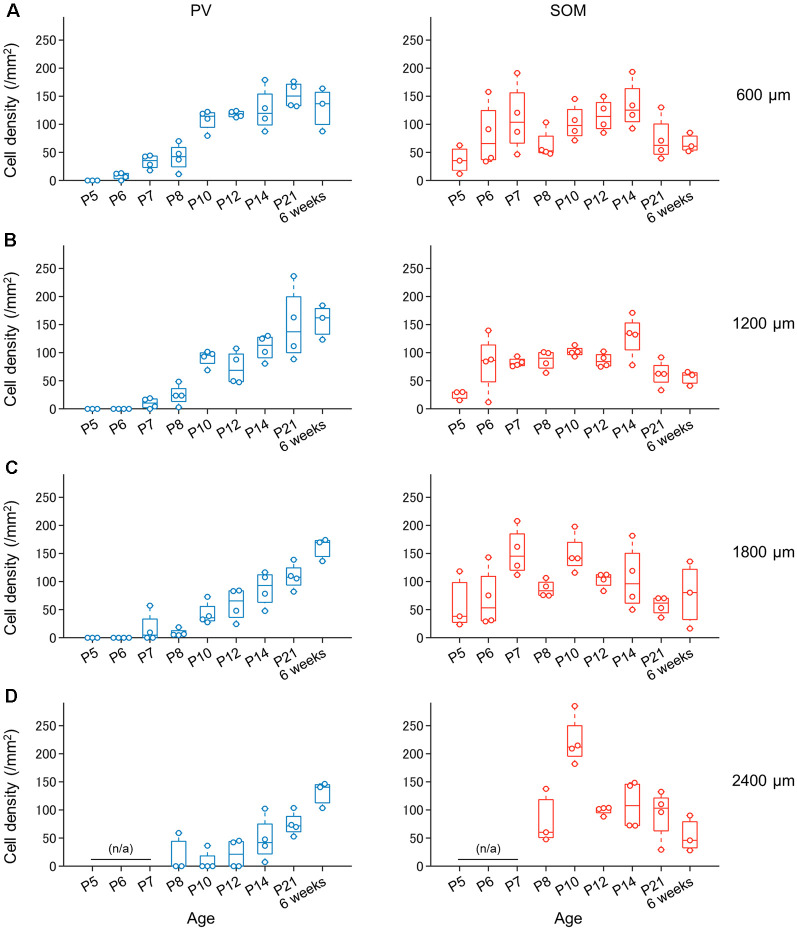
Progressive increase in the number of PV-expressing (not SOM-expressing) interneurons in the superficial layers of the presubiculum during development. **(A)** Density of PV-expressing neurons (*blue*, *left*) and SOM-expressing neurons (*red*, *right*) in the superficial layers of the presubiculum (at 600 μm from the dorsomost level) during development. Note that the number of PV-expressing neurons increases progressively with age, whereas the number of SOM-expressing neurons does not. **(B–D)** The same as **(A)** but at 1,200 μm, 1,800 μm, and 2,400 μm, respectively. PV, parvalbumin; SOM, somatostatin.

**Figure 14:**
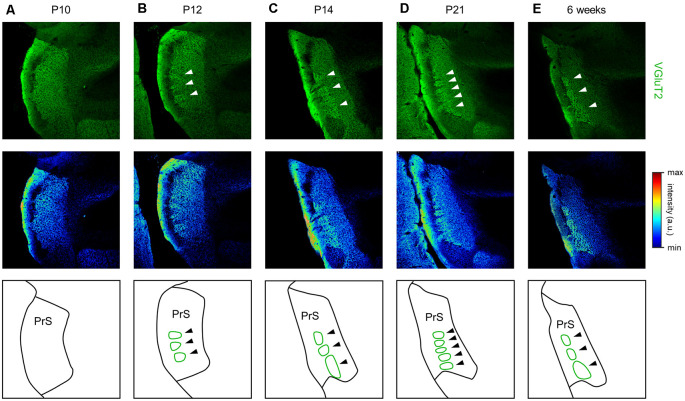
Emergence of VGluT2-positive repeating patch structures in layer III of the presubiculum from 12 day postnatal mouse. **(A)** Representative immunohistochemical image of the presubiculum of a postnatal 10-day-old mouse (*top*). The section was immunostained for VGluT2 (*green*). The *top* image is shown in a pseudocolored manner (*middle*). The VGluT2 immunosignals are almost uniform. The schema of the presubicular superficial layers is presented in the *bottom* panel. **(B)** The same as **(A)** but for a postnatal 12-day-old mouse. At this age, VGluT2 immunosignals in layer III are heterogeneous, as indicated by the *white* arrows. The VGluT2-positive repeating structure is depicted in the schema in the *bottom* panel (*green*). **(C–E)** The same as **(B)** but for postnatal 14-day-old, 21-day-old, and 6-week-old mice, respectively. VGluT2, vesicular glutamate transporter 2; PrS, presubiculum.

Based on this delimitation, we immunostained brain slices from neonatal, juvenile, and adult mice using a VGluT2 antibody and confirmed that VGluT2 immunoreactivity was observed at P5 and ever afterward. We then outlined the VGluT2-positive area to determine the presubicular superficial layers. The area of the superficial layers of the presubiculum decreased from the dorsal to the ventral axis at all ages (Figure 4, Supplementary Figure 4) and was the largest at P14 at most dorsoventral levels (Supplementary Figure 5).

We also found that thalamic axon terminals were absent in layer II of the presubiculum (Figure 5). Based on previous investigations (Boccara et al., 2010; Tukker et al., 2015), we immunostained AAV-injected brain slices using an anti-calbindin antibody and confirmed that calbindin-immunopositive cells form a complementary expression pattern to thalamic axon terminals (Figure 5). Calbindin expression was abundant in layer II in the presubiculum (Figure 5, Supplementary Figure 14), which is consistent with previous research (Preston-Ferrer et al., 2016).

### PV- and SOM-Expressing Interneuronal Density and Repeating Patch Structure in the Developing Presubiculum

We investigated the postnatal changes in the densities of PV-and SOM-expressing interneurons in the presubicular superficial layers. We performed immunostaining of brain slices from P5, P6, P7, P8, P10, P12, P14, P21, and 6-week-old mice using antibodies against VGluT2, PV, and SOM (Figures 6–11, Supplementary Figures 6–9). Consistent with previous research (Nassar et al., 2015), PV-expressing, SOM-expressing, and coexpressing interneurons were all observed in the presubicular superficial layers (Figure 12). Since SOM immunofluorescence was typically vague, we coimmunostained the slices using anti-SOM and anti-GABA antibodies and confirmed that these two signals overlapped, suggesting valid SOM immunostaining (Supplementary Figure 10). We found that SOM-expressing interneurons already existed at P5 while PV-expressing interneurons started to appear at P6. During postnatal development, the cell density of PV-expressing interneurons significantly increased as a function of age at all dorsoventral levels (Figure 13, Supplementary Table 3). In contrast, the density of SOM-expressing interneurons did not exhibit specific increasing trends at either level (Figure 13, Supplementary Table 3).

We further found a repeating patchy pattern of VGluT2-immunopositive fluorescence in slices prepared from adult mice (Figure 14). We further investigated the emergence of this structure during development (Figure 14), and the immunosignals confirmed that the repeating patch structures emerged at P12 and was conserved thereafter.

## Discussion

**Figure 15:**
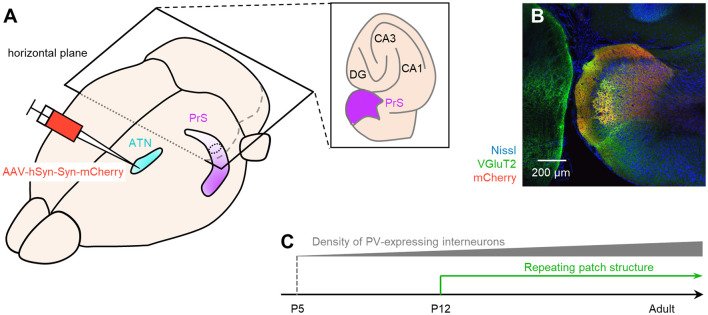
Summary of the current study. **(A)** Bird’s-eye view of the mouse brain. We injected AAV-hSyn-Syn-mCherry (*red*), an anterograde tracer, into the ATN (*pale blue*) and visualized the superficial layers of the presubiculum (*purple*) based on mCherry signals. **(B)** mCherry signals (*red*) in the presubicular superficial layers totally overlapped with the VGluT2 immunosignals (*green*). We then considered that the VGluT2 immunosignals could be used to delimit the presubicular superficial layers during development. **(C)** We coimmunostained VGluT2, PV, and SOM during development and found that the number of PV-positive interneurons gradually increased after P5. We further accidentally found that the VGluT2 immunopositive area in layer III in the presubiculum formed a repeating patch structure from P12. VGluT2, vesicular glutamate transporter 2; PrS, presubiculum; ATN, anterior thalamic nucleus; DG, dentate gyrus; PV, parvalbumin; SOM, somatostatin; AAV, adeno-associated virus.

Here, we identified superficial layers of the presubiculum based on anterograde tracing from the ATN and confirmed that VGluT2 immunosignals overlapped with projection-defined presubicular superficial layers. Using VGluT2 immunosignals, we found that the presubicular superficial layers were larger in the dorsal region than in the ventral region at all ages. Moreover, the number of PV-expressing interneurons increased progressively with age at all dorsoventral (i.e., dorsal-to-ventral) levels (Figure 15), whereas SOM-expressing interneurons did not exhibit any specific increasing trend in number. Furthermore, the VGluT2 immunosignals constituted a patch structure in presubicular layer III. The spatially clustered patch structure appeared during adolescence (Figure 15).

### Utility of VGluT2 for Anatomical Characterization of the Presubiculum

VGluT1 and VGluT2 proteins are usually present primarily in axon terminals of glutamatergic excitatory neurons to incorporate glutamate into synaptic vesicles. The two isoforms generally display an almost complementary distribution in the brain (Kaneko et al., 2002; Fremeau et al., 2004b). In the ATN, however, neuronal somata exhibit both VGluT2 and VGluT1 immunoreactivities (Oda et al., 2014; Supplementary Figure 11), which is consistent with the colocalization of mRNA encoding VGluT2 with VGluT1 mRNA (Herzog et al., 2001; Barroso-Chinea et al., 2007). Based on a previous anatomical investigation (Kaneko et al., 2002), we immunostained coronal sections for VGluT2 and delineated the ATN, including the anterior dorsal thalamic nucleus (AD) and the anterior ventral thalamic nucleus (AV), to confirm the injection site (Figure 1B). Note that there is a pattern in which the VGluT2 immunofluorescence intensity in AD is slightly stronger than that in AV, which is consistent with previous research (Oda et al., 2014; Supplementary Figure 2).

We previously located the VGluT2-immunoreactive region downstream of the subiculum (Kashima et al., 2019), as suggested by others (Wouterlood et al., 2008; Ishihara and Fukuda, 2016). Nevertheless, no study has empirically characterized the VGluT2-immunopositive area in the presubiculum, whereas the VGluT2 expression pattern in the hippocampus has already been investigated (Halasy et al., 2004). Based on previous anatomical investigations (Robertson and Kaitz, 1981; Shibata, 1993; Nassar et al., 2018; Mathiasen et al., 2020), we injected an anterograde tracer into the ATN (subdivided into the AD and AV) and labeled the superficial layers of the presubiculum. Consistent with a previous report (Nassar et al., 2018), we confirmed that this anterogradely labeled area was VGluT2 immunopositive and utilized VGluT2 to characterize the presubicular superficial area in this study. On the contrary, the immunoreactivity of VGluT1 was much weaker in the presubicular superficial layers compared with that of VGluT2 (Supplementary Figure 12).

### Thalamic Axon Terminals Expressing VGluT2 Form Putative Excitatory Synapses Impinging Onto Dendrites in the Presubicular Superficial Layers

We coimmunostained brain slices from AAV-injected mice using an anti-PSD95 (postsynaptic density 95) antibody to confirm that the mCherry signals were putative presynaptic terminals (Sheng and Kim, 2011; Villa et al., 2016). The mCherry signals were proximately apposed to PSD95 immunosignals, but the two signals did not overlap, suggesting that mCherry labeling signifies excitatory presynaptic terminals (Supplementary Figure 13). Additionally, putative excitatory synapses from thalamic axons onto the presubicular superficial layers were unevenly distributed. The mCherry signals indicative of thalamic terminals were more enriched in layers I and III than in layer II (Figure 1), suggesting that anterior thalamic axons are unlikely to target layer II in the presubiculum. Most mCherry signals were located close to the PSD95 signals beside MAP2 (microtubule-associated protein 2) signals (Supplementary Figure 13); note that MAP2 isoforms are neuron-specific cytoskeletal proteins and abundant in dendrites and perikarya. Similar to the mCherry signals, VGluT2 signals were next to the MAP2 signals (Supplementary Figure 13). In contrast, the PSD95 signals were hardly seen in layer I but are obvious in layers II and III (Supplementary Figure 14). Overall, we concluded that most putative ATN-to-presubicular excitatory synapses are formed mainly on dendrites in layer III in the presubiculum. However, we could not precisely localize the cell bodies of the presubicular neurons, because both excitatory and inhibitory neurons in multiple layers in the presubiculum extend their dendrites into layer III (Simonnet et al., 2013; Nassar et al., 2015).

Using slices from three AAV-injected mice, we calculated the ratio of *N_VGluT2(+)_and_ mCherry(+)_* to *N_mCherry(+)_*, where *N_criteria_* signified the number of boutons (puncta) that satisfied the criteria (Supplementary Figure 3). The ratios of the VGluT2-positive terminals from three mice were 93.6%, 94.8%, and 96.4%. Therefore, we consider that almost all thalamic axon terminals in the presubiculum contain VGluT2. Conversely, we observed VGluT2 signals that were not colocalized with the mCherry signals. The superficial layers in the presubiculum are innervated by neurons in not only the ATN (Nassar et al., 2018) but also the subiculum (Naber and Witter, 1998), the parasubiculum (van Groen and Wyss, 1990a), the entorhinal cortex (van Groen and Wyss, 1990a), and the retrosplenial cortex (Jones and Witter, 2007; Sugar and Witter, 2016). Since axonal terminals from the entorhinal cortex to the hippocampal CA1 area are VGluT1-positive (Kitamura et al., 2014), it is less likely that the entorhinal cortex is the source region of the VGluT2-positive axonal terminals. To the best of our knowledge, however, whether axonal terminals from the subiculum, the parasubiculum, and the retrosplenial cortex are immunopositive for VGluT1 or VGluT2 is uncertain. Thus, we just speculate that the origins of VGluT2-positive axonal terminals are the subiculum, the parasubiculum, and the retrosplenial cortex, except for the ATN.

### Implications of VGluT2 Immunosignals for Synaptic Functions

It is still controversial whether the transport activities of VGluT1 and VGluT2 are different (Bellocchio et al., 2000; Takamori et al., 2000, 2001; Fremeau et al., 2001; Zhang et al., 2018); however, the expression of the two isoforms is likely to be differentially associated with synaptic strength and synaptic plasticity. VGluT1 and VGluT2 are correlated with a low and high release probability (*P_r_*) of neurotransmitters, respectively (Fremeau et al., 2001, 2004a). For example, VGluT1 is abundant in the hippocampal *stratum radiatum*, where Schaffer collaterals terminate (Kaneko et al., 2002; Fremeau et al., 2004a; Jung et al., 2018). At Schaffer collateral-CA1 synapses, *P_r_* is mostly between 0.1 and 0.8 (Hessler et al., 1993; Larkman et al., 1997; Branco and Staras, 2009). On the other hand, VGluT2 localizes in climbing fiber boutons in the molecular layer in the cerebellum (Fremeau et al., 2001), and the *P_r_* at cerebellar climbing fiber synapses is approximately 0.9 (Silver et al., 1998; Branco and Staras, 2009).

Moreover, VGluT1 and VGluT2 are differentially involved in synaptic plasticity. With respect to short-term synaptic plasticity, *in vitro* electrophysiology of acute hippocampal slices prepared from homozygous VGluT1 knockout mice has confirmed that fast recovery from synaptic fatigue caused by repetitive stimulation necessitates VGluT1 (Fremeau et al., 2004a). Regarding long–term plasticity, VGluT1 is required for long–term potentiation in the hippocampus (Balschun et al., 2010) while VGluT2 deficiency impairs long–term depression in the young hippocampus (He et al., 2012), although it is less abundant in the hippocampus than VGluT1.

Collectively, the abundant expression of VGluT2 suggests that presubicular neurons receive high-fidelity neurotransmission, which may contribute to a pathway that is more hard-wired than VGluT1-abundant Schaffer collateral-CA1 synapses (Varoqui et al., 2002). Thus, in light of the VGluT expression pattern, we speculate that the presubiculum plays a distinct role in terms of synaptic function compared with the hippocampus proper. Moreover, the presubiculum is indeed as pivotal a region for spatial exploration as the hippocampus; however, the presubicular and hippocampal representations for space are distinct, as further discussed below.

### Functional Relevance to Spatial Representation

We found that clustered high VGluT2 immunofluorescence constituted repeating patch structures, especially in layer III, in the dorsal presubiculum of P12 and older mice (Figure 14). A previous study reported a vertical columnar structure similar to the repeating patch structures found in our study (Nishikawa et al., 2002); however, the two types of structures are definitely different in three aspects: (1) age; (2) neuronal substrate; and (3) sectional plane. (1) In our study, repeating patch structures that emerge from P12 are never seen before the age, whereas the vertical columns emerge at P2–P4 and become hardly discernible from P14. (2) The patch structures (in the current study) are visible by VGluT2 immunostaining, not by Nissl staining. On the other hand, the vertical columns are found based on Nissl staining, thus forming a complementary pattern with Cajal-Retzius cells labeled by immunostaining using CR50 (i.e., a mouse monoclonal antibody that recognizes an epitope in the N-terminal region of reelin). (3) The repeating patch structures and the vertical columnar structure are observed on horizontal and coronal planes, respectively.

Similar structures were also previously reported in the visual cortex (Ichinohe et al., 2003) and the medial entorhinal cortex (Burgalossi et al., 2011; Ray et al., 2014; Ebbesen et al., 2016; Naumann et al., 2016, 2018; Ray and Brecht, 2016; Tang et al., 2016); however, all of these structures were calbindin positive and found in layer II. Although we found that layer II in the presubiculum was calbindin positive, which was consistent with previous studies (Boccara et al., 2010; Tukker et al., 2015), we did not find any small patches in layer II. Instead, we found VGluT2-immunofluorescent patches, suggesting that anterothalamic excitatory innervations may unevenly converge onto the presubicular superficial layers.

In the presubiculum, the most intriguing neural correlates of spatial behavior are head-direction cells (Taube et al., 1990a, b; Taube, 2007; Yoder et al., 2011; Gibson et al., 2013; Tukker et al., 2015), which are also found in AD (Taube, 1995). An experimental model suggests that the head-direction signal in AD streams to the presubiculum (Taube, 2007). We thus conjecture that the presubicular layer III neurons inside the VGluT2-positive patches receive more head-directional inputs from AD than those outside and might exhibit massive direction-selective firing. In layer III of the presubiculum, pyramidal cells are divided into two types in terms of head-directional selectivity firing; that is, firing of almost 40% of the pyramidal cells recorded is strongly modulated by head-direction, whereas the others exhibit moderate direction-selective firing (Tukker et al., 2015). Collectively, presubicular neurons inside the VGluT2 patches may receive numerous synaptic inputs from the anterior thalamus and thus exhibit strong direction-selective firing.

Interestingly, mCherry (in layer III) and calbindin (in layer II) displayed an almost complementary expression pattern in the presubicular superficial layers (Figure 5), consistent with previous research reporting that layer II in the presubiculum is calbindin positive (Boccara et al., 2010; Tukker et al., 2015). Nevertheless, the firing properties of calbindin-positive neurons in layer II in the presubiculum are elusive. In layer II of the medial entorhinal cortex, calbindin-positive pyramidal cells form patches at regular intervals and exhibit stronger theta-rhythmicity of spiking than calbindin-negative cells (Ray et al., 2014). As with the entorhinal cortex, we assume that the presubicular calbindin-positive neurons in layer II emit firing modulated by theta rhythms, opposed to the limited theta rhythmicity of spiking in presubicular pyramidal cells in layer III (Tukker et al., 2015).

A long line of studies has proposed that input of vestibular information into the ATN is necessary for the generation of head-direction signals (Taube, 2007), which emerge at P12–P15 (Langston et al., 2010; Bjerknes et al., 2015). Once generated, maintenance of head-direction signals of the presubicular pyramidal cells in adult mice requires feedback inhibition from SOM-positive neurons (i.e., Martinotti cells) dependent on pyramidal cell firing (Simonnet et al., 2017). The adult-like directional signals during the early period and the activity-dependent feedback inhibition during adulthood seem to contradict the age-irrelevant number of SOM-positive interneurons found in this study. However, this contradiction may be reconciled by the immaturity of the membrane properties and the excitability of SOM-positive interneurons during the neonatal and juvenile periods; note that this postnatal maturation has been investigated in layers II/III of the secondary motor cortex, not the presubiculum (Pan et al., 2019).

In addition to the head-direction selectivity of firing, presubicular neurons discharge at regular intervals when an animal actively explores an open field (Boccara et al., 2010). Such spatially tuned neurons are called grid cells, and they have been investigated extensively in the medial entorhinal cortex (Fyhn et al., 2004, 2007; Hafting et al., 2005, 2008; Derdikman and Moser, 2010; Stensola et al., 2012, 2015; Moser et al., 2013; Wernle et al., 2018; Hägglund et al., 2019) as well as in the presubiculum and parasubiculum (Boccara et al., 2010). Robust grid-like representation appears in the medial entorhinal cortex almost at P20 (Langston et al., 2010; Wills et al., 2010). Moreover, pharmacogenetic inactivation of PV-positive (but not SOM-positive) interneurons in the medial entorhinal cortex and parasubiculum impairs spatial periodicity in grid cells (Miao et al., 2017). PV-expressing neurons also provide entorhinal grid cells with recurrent inhibition (Buetfering et al., 2014). Based on the similarity in physiological functions between the presubiculum and medial entorhinal cortex, we speculate that a progressively increasing number of PV-positive neurons with age may be involved in the development of grid codes.

### Concluding Remarks

Stellate cells and pyramidal cells are abundant in layers II and III of the presubiculum, respectively (Funahashi and Stewart, 1997a, b; Simonnet et al., 2013; Peng et al., 2017; Simonnet and Fricker, 2018). The current immunohistochemical investigation also confirmed that presubicular layers II and III are segregated based on calbindin and VGluT2 expression. Moreover, we found a novel type of VGluT2-immunoreactive mosaic patch structure in layer III. Electrophysiology followed by *post hoc* immunohistochemistry and single-cell labeling (e.g., whole-cell, intracellular, and juxtacellular recording techniques) can identify cell type-and layer-specific neuronal functions and thereby reveal the relationship between anatomy and physiology.

Moreover, using the VGluT2-based definition, we evaluated the interneurons in the superficial layer of the presubiculum along with their development in terms of the number; however, the postnatal maturation of physiological functions of the interneurons is still unclear. With the aid of VGluT2 immunohistochemistry, future *in vivo* electrophysiological experiments on the developing presubiculum with genetic manipulation will shed light upon the functional relevance of interneuronal maturation to spatial representation and unveil new aspects of the presubiculum as a unique compartment distinct from the hippocampus.

## Data Availability Statement

The datasets presented in this study can be found in online repositories. The names of the repository/repositories and accession number(s) can be found below: https://bit.ly/3rggl4N.

## Ethics Statement

The animal study was reviewed and approved by the Animal Experiment Ethics Committee of the University of Tokyo.

## Author Contributions

YI and NM conceptualized the research. JL, TK, SM, and AN performed the experiments. SM provided materials. JL and NM analyzed the data. JL, TK, SM, AN, YI, and NM discussed the project and wrote the manuscript by mutual consent. All authors contributed to the article and approved the submitted version.

## Conflict of Interest

The authors declare that the research was conducted in the absence of any commercial or financial relationships that could be construed as a potential conflict of interest.
