# Supplementary material for: Molecular Characterization of Superficial Layers of the Presubiculum During Development
Source: Front Neuroanat. 2021 May 24;15:662724. doi: 10.3389/fnana.2021.662724 (PMC8256428; doi:10.3389/fnana.2021.662724)
Supplement: Supplementary file 1 [file Data_Sheet_1.PDF]

Liu *et al.*, Supplementary Materials

Contents

- Supplementary Figure 1
  Horizontal section of the mouse brain.
  Page 1
- Supplementary Figure 2
  VGluT2 immunoreactivity and AAV-mediated mCherry expression in thalamic injection sites and thalamic axonal target sites.
  Pages 2-3
- Supplementary Figure 3
  Representative photographs of VGluT2 immunoreactivity and AAV-mediated anterograde tracing in superficial layers of the presubiculum.
  Page 4
- Supplementary Figure 4
  Dorsal-to-ventral decrease in the area of the presubicular superficial layers across all ages.
  Page 5
- Supplementary Figure 5
  Age dependency of the area of the presubicular superficial layers at each dorsoventral level.
  Page 6
- Supplementary Figure 6
  PV and SOM immunoreactivity in the superficial layers of the presubiculum across ages.
  Pages 7-8
- Supplementary Figure 7
  Representative photographs of the presubicular superficial layers of a postnatal 7-day-old mouse.
  Page 9
- Supplementary Figure 8
  Representative photographs of the presubicular superficial layers of a postnatal 8-day-old mouse.
  Page 10
- Supplementary Figure 9
  Representative photographs of the presubicular superficial layers of a postnatal 14-day-old mouse.
  Page 11
- Supplementary Figure 10
  SOM-immunopositive presubicular neurons express GABA.
  Page 12
- Supplementary Figure 11
  Expression of VGluT1 and VGluT2 in the thalamic injection site.
  Page 13
- Supplementary Figure 12
  Few VGluT1 expression in the presubicular superficial layers.
  Page 14
- Supplementary Figure 13
  Thalamic axon terminals forming putative excitatory synapses impinging onto dendrites in the presubicular superficial layers.
  Page 15
- Supplementary Figure 14
  Distribution of putative synapses from thalamic axons onto neurons in the presubicular superficial layers.
  Page 16
- Supplementary Table 1
  Representative values of the spatial correlations.
  Page 17
- Supplementary Table 2
  Statistics for the spatial correlations.
  Page 18
- Supplementary Table 3
  Statistics for the age-dependent cell density of interneurons.
  Page 19

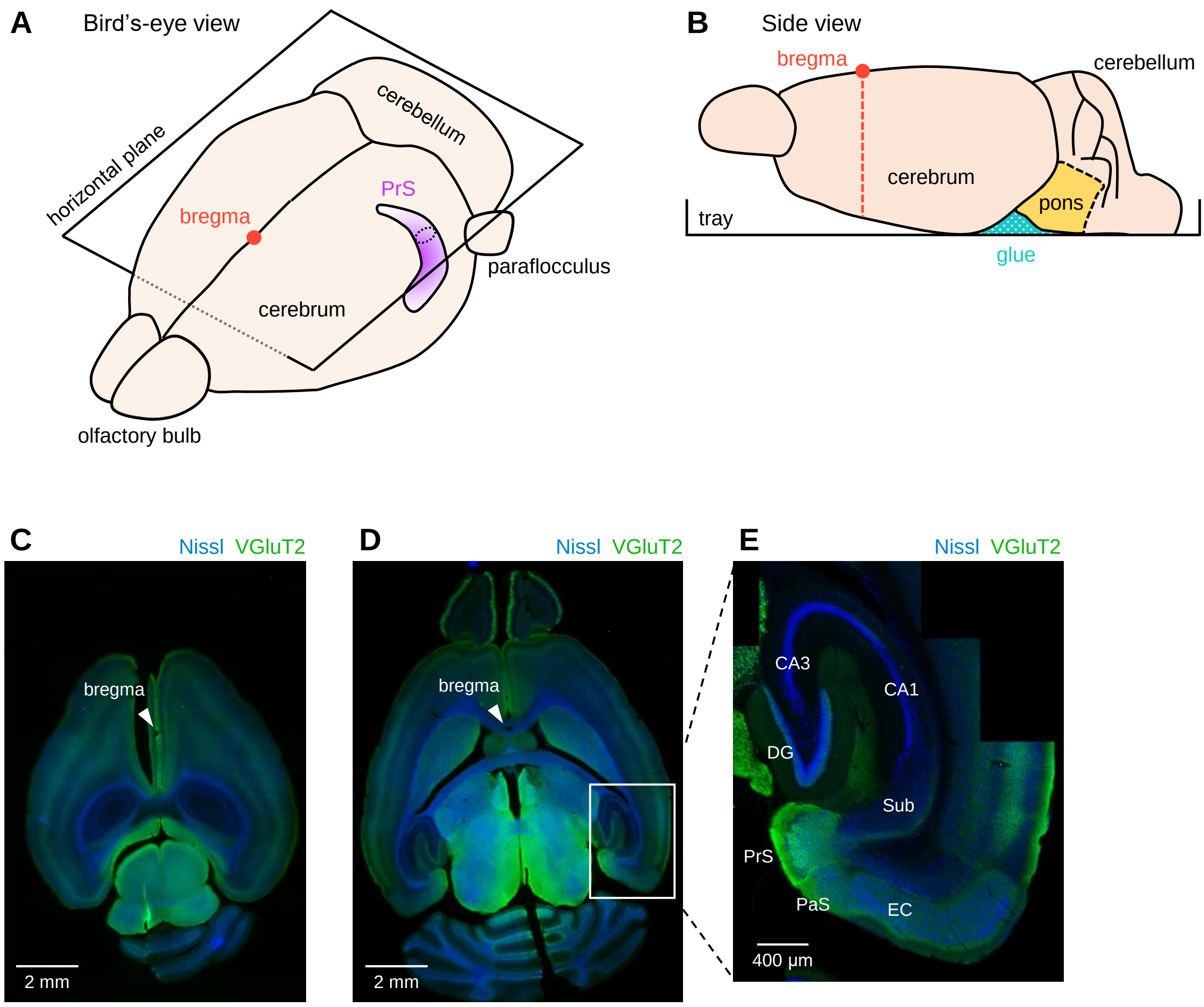

Supplementary Figure 1 | Horizontal sectioning of the mouse brain.
A, Bird's-eye view of the mouse brain. The brain is horizontally sectioned. ***B***, Side view of the mouse brain, which was fixed to the tray with glue (*light blue*) attached to the connection between the cerebrum and the pons (*orange*). We vertically pricked the brain from bregma (*red*) using a needle to investigate the anterior-to-posterior coordinate of bregma in horizontal sections (***C***, ***D***). ***C***, Low-magnification image of the horizontal section, which is defined as the 'dorsal tip' in this study. The coordinate of the dorsal tip (*i.e.*, zero-reference) along the dorsal-to-ventral axis was approximately 1400 μm beneath the brain surface. The slice was stained for Nissl (*blue*) and immunostained for VGluT2 (*green*). The location of bregma is indicated by a *white* arrow. ***D***, The same as ***B***, but for the slice at 1200 μm from the dorsal tip. ***E***, Magnified image of the boxed area in ***C***. *Abbreviations*: VGluT2, vesicular glutamate transporter 2; DG, dentate gyrus; Sub, subiculum; PrS, presubiculum; PaS, parasubiculum; EC, entorhinal cortex.

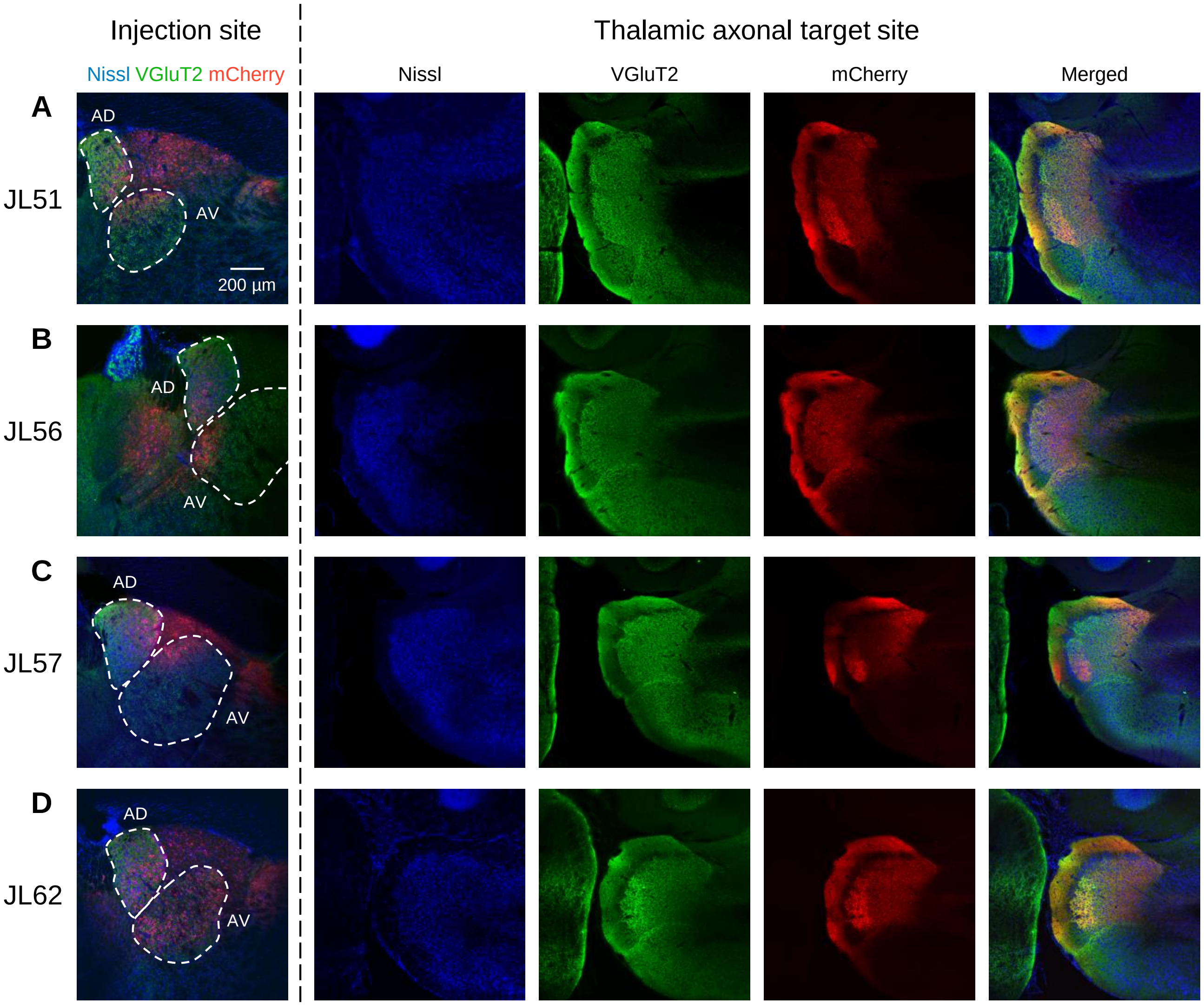

Supplementary Figure 2 | VGluT2 immunoreactivity and AAV-mediated mCherry expression in thalamic injection sites and thalamic axonal target sites.
A, Representative photographs of the sections of the AAV-injected mouse named JL51. Coronal sections of the injection site (*leftmost* (*first*)) were stained for Nissl substances (*blue*), immunostained for VGluT2 (*green*), and simultaneously visualized with AAV-mediated mCherry (*red*). The regions of AD and AV are encircled by *white* dashed lines. Horizontal sections of the thalamic axonal target sites (*i.e.*, the superficial layers of the presubiculum) were stained for Nissl substances (*blue*, *second*), immunostained for VGluT2 (*green*, *third*), and simultaneously visualized with AAV-mediated anterograde tracing (mCherry; *red*, *fourth*). A merged image is displayed in the *fifth* column. ***B-F***, The same as ***A***, but for mice named JL56, JL57, JL62, JL64, and JL65, respectively. *Abbreviations*: AAV, adeno-associated virus; VGluT2, vesicular glutamate transporter 2; AD, anterior dorsal thalamus; AV, anterior ventral thalamus.

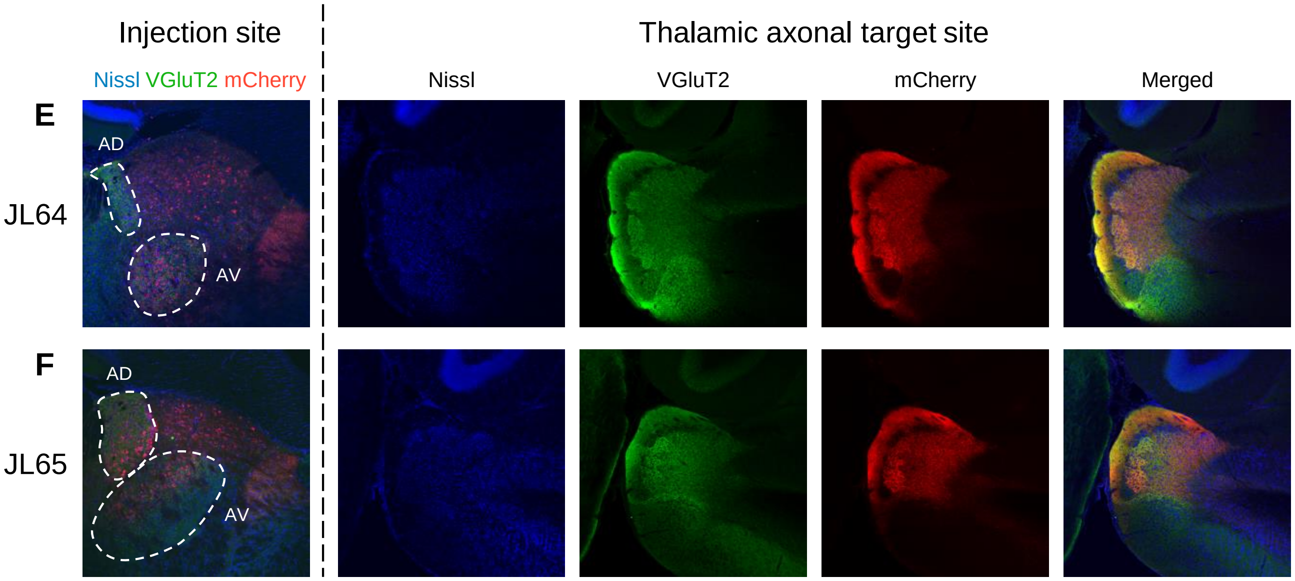

Supplementary Figure 2 | VGluT2 immunoreactivity and AAV-mediated mCherry expression in thalamic injection sites and thalamic axonal target sites.
Continued from the previous page.

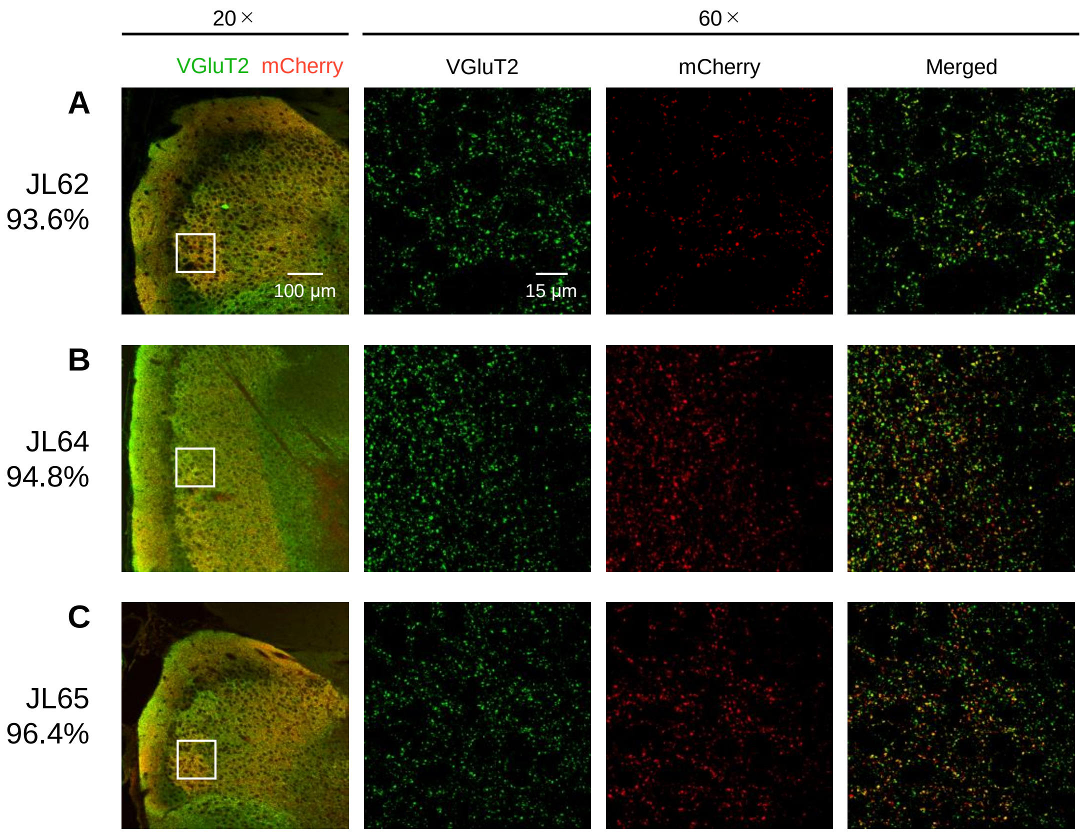

Supplementary Figure 3 | Representative photographs of VGluT2 immunoreactivity and AAV-mediated anterograde tracing in superficial layers of the presubiculum.

A, Confocal images (20× objective) of the presubicular superficial layers (*leftmost* (*first*)) of a mouse named JL62. VGluT2 (*green*) and mCherry (*red*) signals are displayed. Magnified images (60× objective and 2× electronic zoom) of the boxed area in the *first* panel are shown in the *second*, *third*, and *fourth* panels. The images of VGluT2 immunosignals (*green*, *second*) and mCherry signals (*red*, *third*) plus the merged image (*fourth*) are displayed. The percentage beneath the mouse's name signifies the proportion of $N_{VGluT2(+)\_and\_mCherry(+)}$ to $N_{mCherry(+)}$, where $N_{criteria}$ signified the number of boutons that satisfied the criteria. ***B-C***, The same as ***A***, but for mice named JL64 and JL65, respectively. *Abbreviations*: AAV, adeno-associated virus; VGluT2, vesicular glutamate transporter 2.

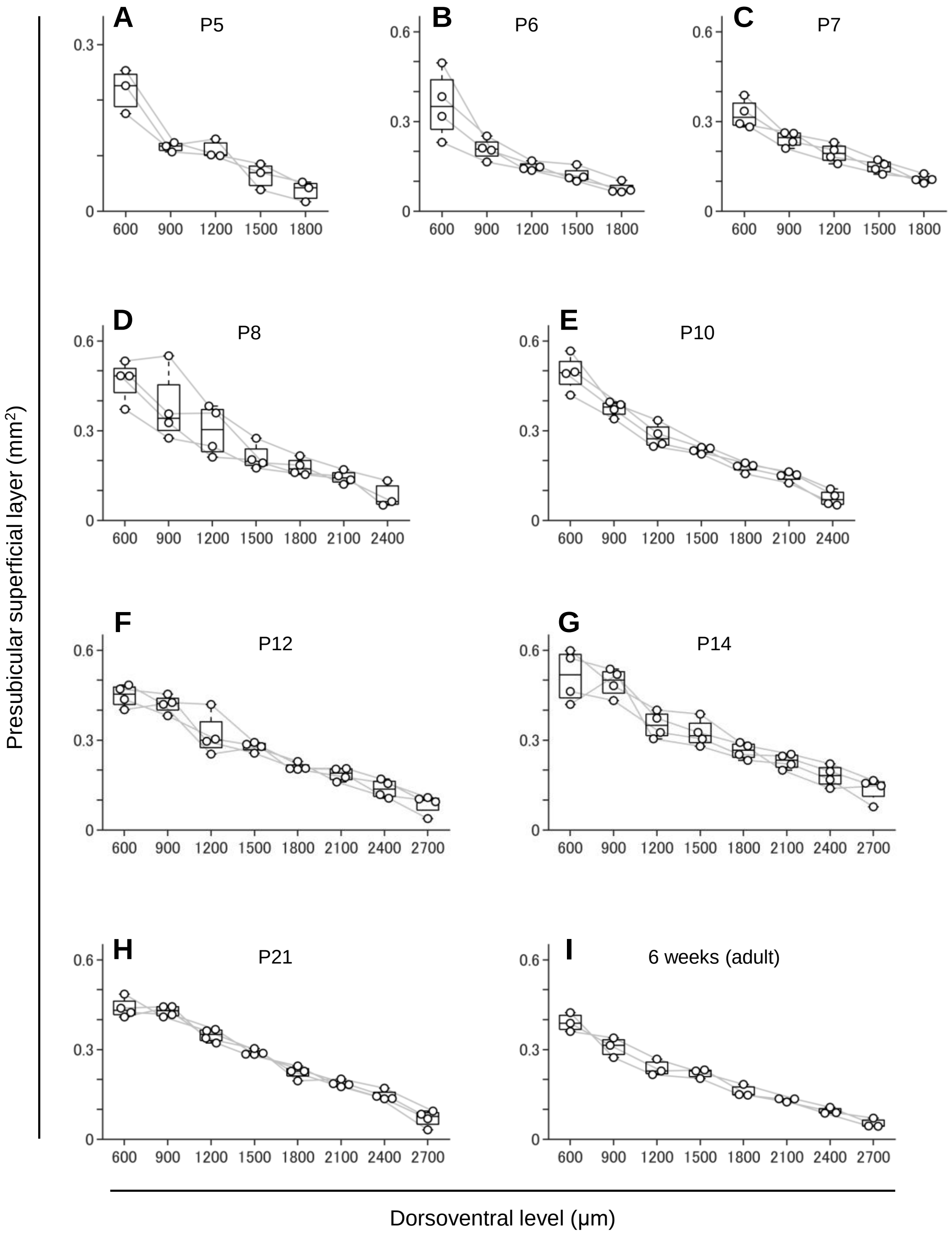

Supplementary Figure 4 | Dorsal-to-ventral decrease in the area of the presubicular superficial layers across all ages.

A, Superficial layers of the presubiculum of P5 mice were determined based on VGluT2 immunofluorescence. The area of the superficial layers was estimated every 300 µm from 600 µm; note that the most dorsal end was defined as 0 µm. The area tended to decrease from the dorsal to ventral end. ***B-I***, The same as ***A***, but for P6, P7, P8, P10, P12, P14, P21, and 6-week-old mice, respectively.

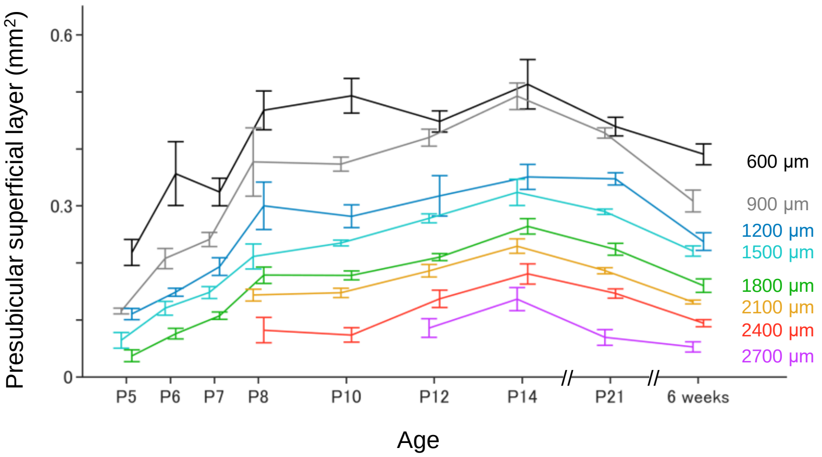

Supplementary Figure 5 | Age dependency of the area of the presubicular superficial layers at each dorsoventral level.

The data presented as the mean ± SEM. Each dorsoventral level is indicated by color (from the most dorsal section: 600 µm, *black*; 900 µm, *gray*; 1200 µm, *blue*; 1500 µm, *light blue*; 1800 µm, *green*; 2100 µm, *yellow*; 2400 µm, *red*; and 2700 µm, *purple*).

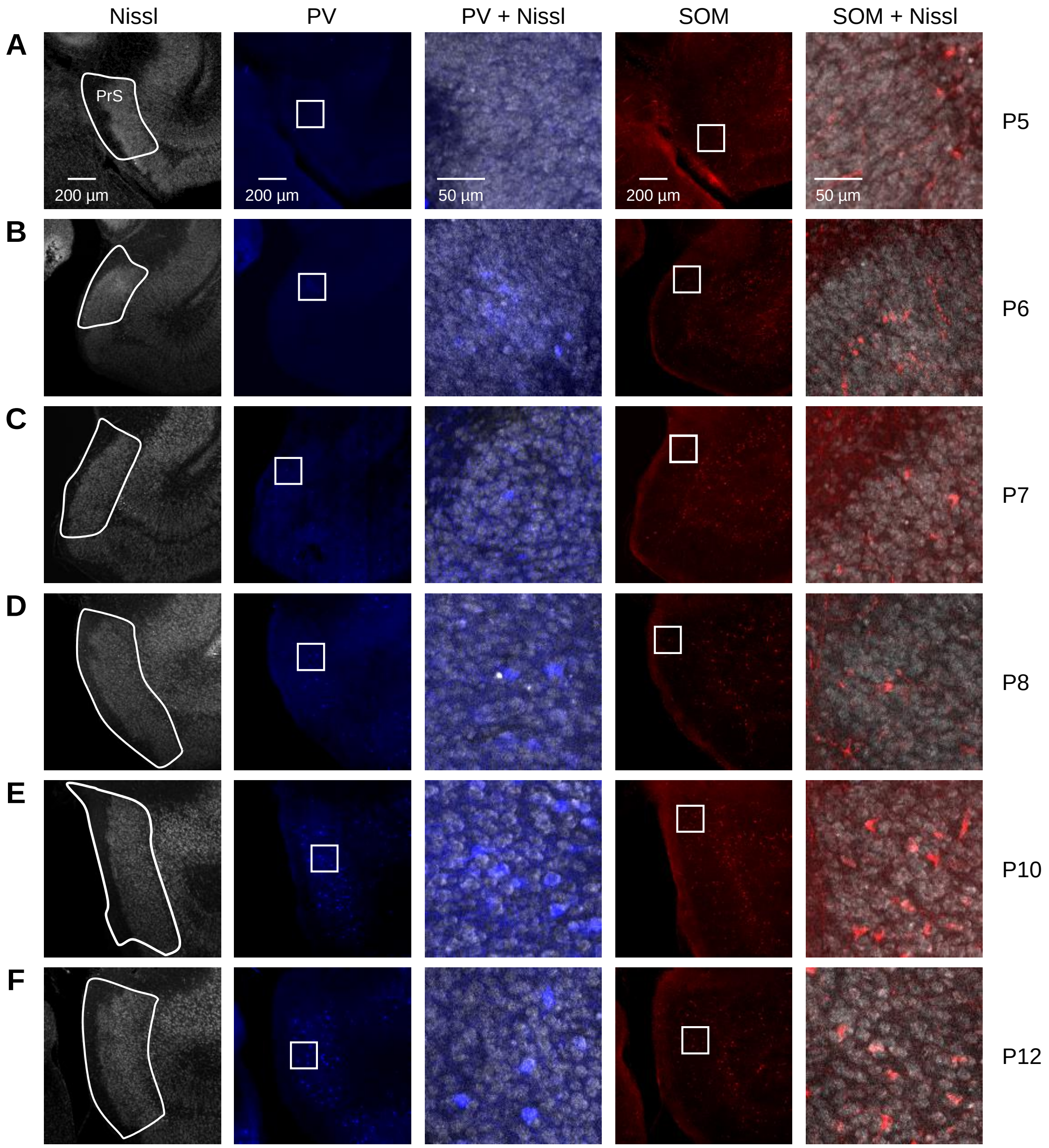

Supplementary Figure 6 | PV and SOM immunoreactivity in the superficial layers of the presubiculum across ages.

A, Representative images of Nissl-stained sections from postnatal 5-day-old (P5) mice (*gray*, *leftmost* (*first*)). The same section was immunostained using antibodies against PV (*blue*, *second* and *third*) and SOM (*red*, *fourth* and *fifth*). The *white* boxed areas in the *second* and *fourth* panels are magnified and displayed in the *third* and *fifth* panels, respectively. The images in the *third* and *fifth* panels were created by merging the Nissl-stained image with the PV-immunostained and SOM-immunostained images, respectively. ***B*–*I***, The same as ***A***, but for P6, P7, P8, P10, P12, P14, P21, and 6-week-old mice, respectively. *Abbreviations*: PV, parvalbumin; SOM, somatostatin.

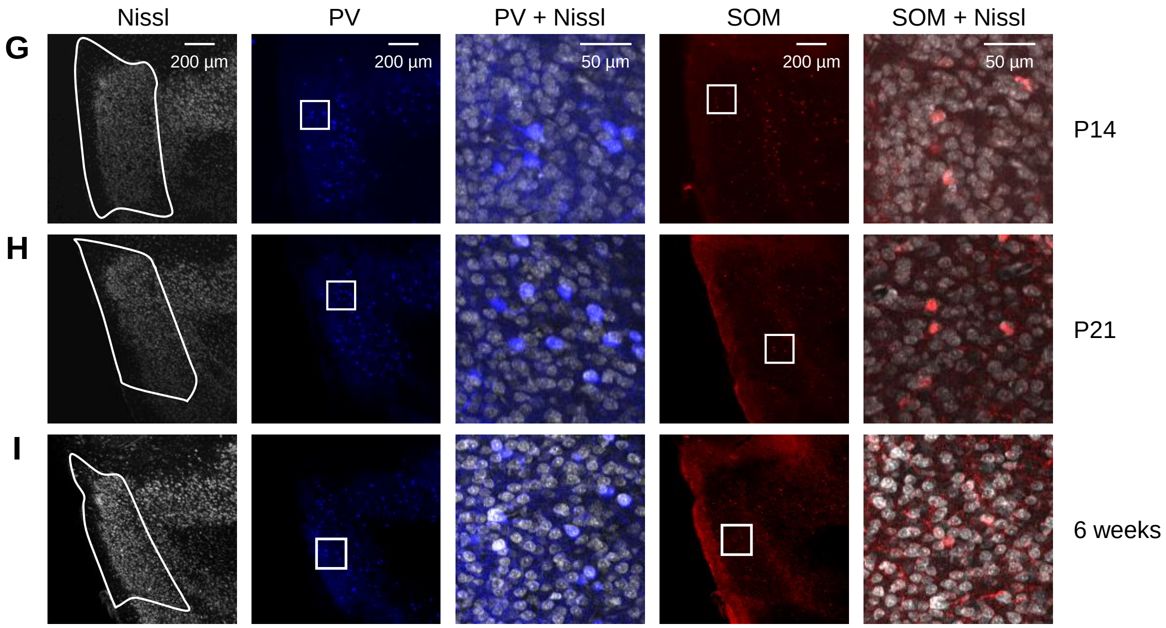

Supplementary Figure 6 | PV and SOM immunoreactivity in the superficial layers of the presubiculum across ages.
Continued from the previous page.

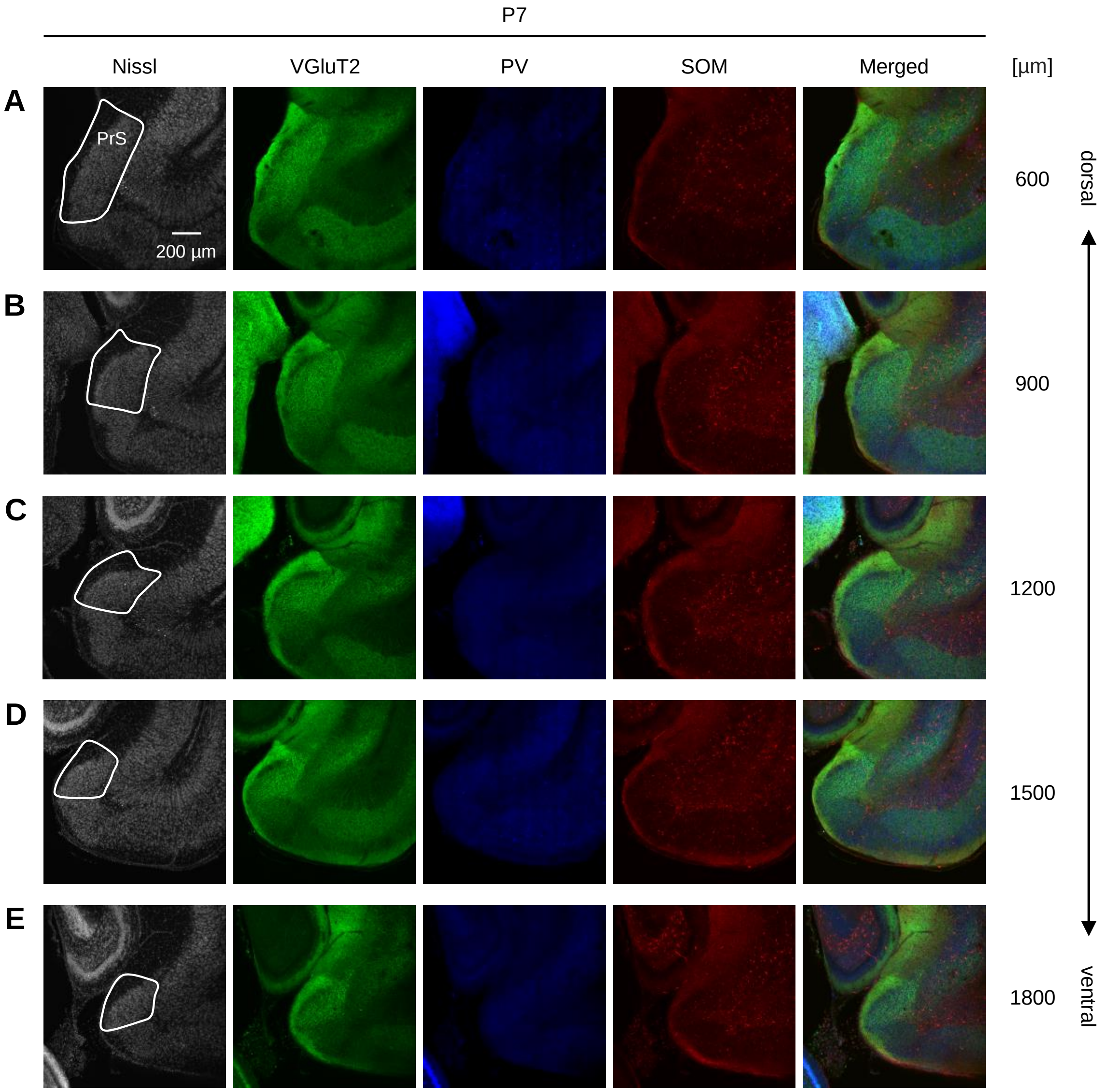

Supplementary Figure 7 | Representative photographs of the presubicular superficial layers of a postnatal 7-day-old mouse.

A, Superficial layers of the presubiculum of a postnatal 7-day-old mouse were stained for Nissl substances (*gray*, *leftmost* (*first*)) and immunostained for VGluT2 (*green*, *second*), parvalbumin (PV; *blue*, *third*), and somatostatin (SOM; *red*, *fourth*) in a slice at 600 µm depth. A merged image (except for Nissl) is displayed in the *fifth* panel. The presubicular superficial layers are delineated by a *white* loop in the *first* panel. The dorsoventral level is indicated as the distance (µm) from the most dorsal section (*i.e.*, 0 µm). ***B-E***, The same as ***A***, but at 900 µm, 1200 µm, 1500 µm, and 1800 µm, respectively. *Abbreviations*: PrS, presubiculum; VGluT2, vesicular glutamate transporter 2; PV, parvalbumin; SOM, somatostatin.

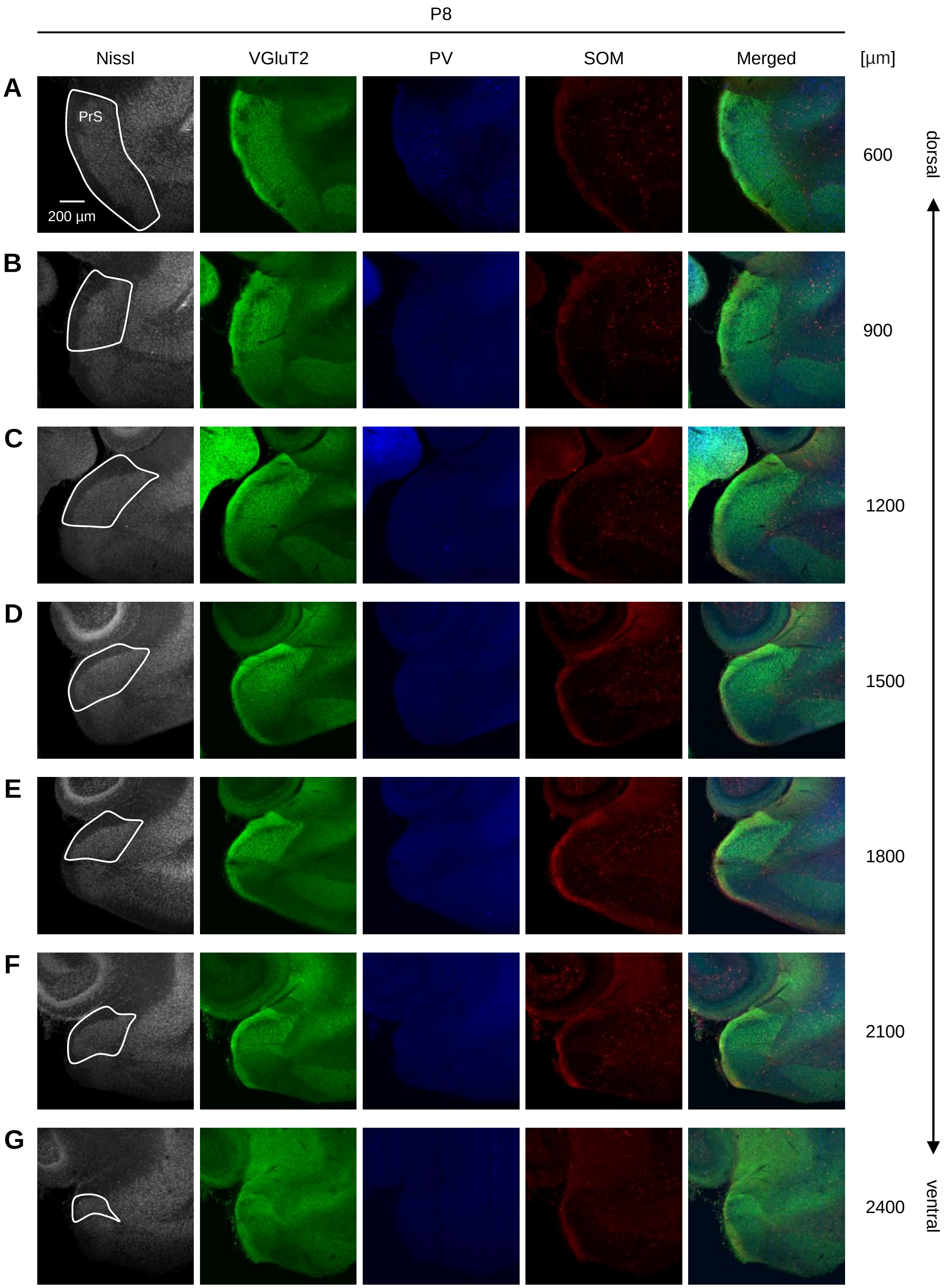

Supplementary Figure 8 | Representative photographs of the presubicular superficial layers of a postnatal 8-day-old mouse.

The same as Supplementary Figure 7 but for a postnatal 8-day-old mouse. ***A*-*G***, Superficial layers of the presubiculum of sections from 600 µm, 900 µm, 1200 µm, 1500 µm, 1800 µm, 2100 µm, and 2400 µm from the most dorsal section (*i.e.*, 0 µm), respectively.

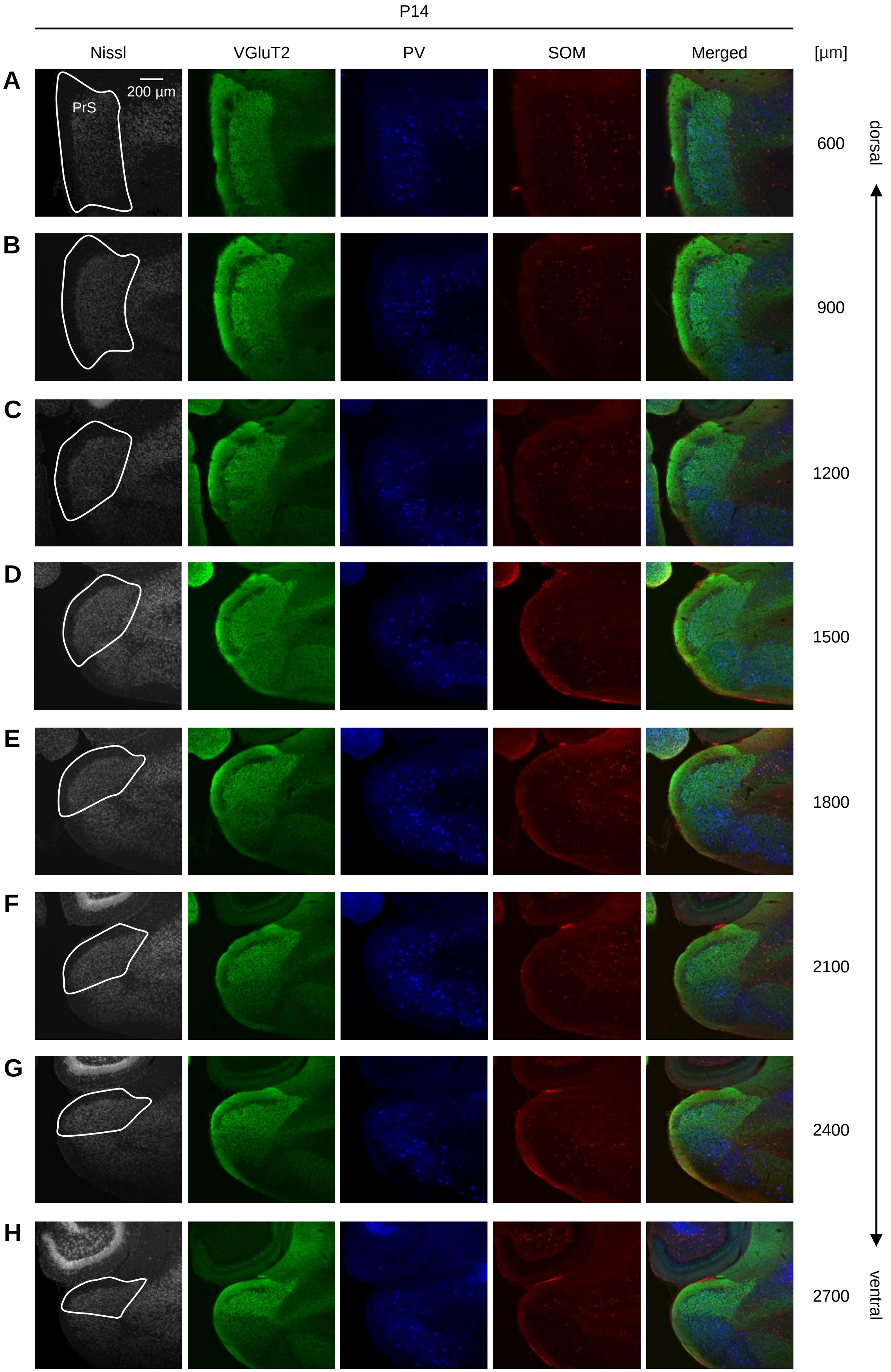

Supplementary Figure 9 | Representative photographs of the presubicular superficial layers of a postnatal 14-day-old mouse.

The same as Supplementary Figure 7, but for a postnatal 14-day-old mouse. ***A-H***, Superficial layers of the presubiculum of sections from 600 µm, 900 µm, 1200 µm, 1500 µm, 1800 µm, 2100 µm, 2400 µm, and 2700 µm from the most dorsal section (*i.e.*, 0 µm), respectively.

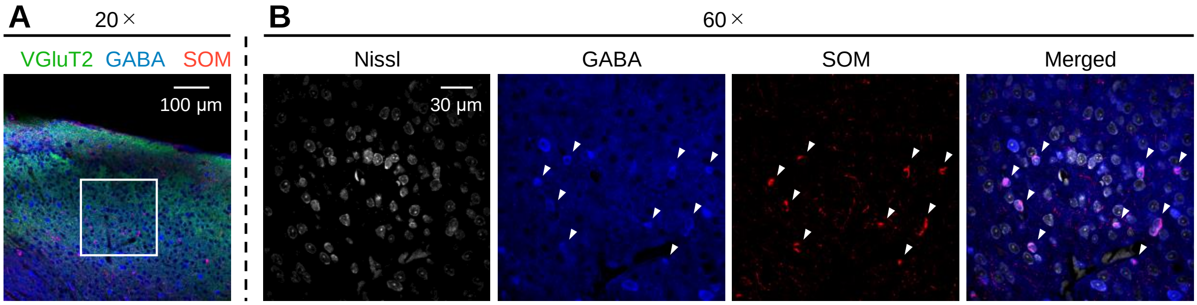

Supplementary Figure 10 | SOM-immunopositive presubicular neurons express GABA.
A, Representative image (20×) of the presubicular superficial layers of an adult mouse (6 weeks old). The section is immunostained for VGluT2 (*green*), GABA (*blue*), and SOM (*red*). ***B***, High-magnification (60×) images of the *white* boxed area in ***A***. The section is stained for Nissl substances (*gray*, *leftmost* (*first*)) and immunostained for GABA (*blue*, *second*) and SOM (*red*, *third*). The merged image is displayed in the *fourth* panel, which shows that the SOM-immunopositive neurons in the presubiculum are also confirmed by GABA immunosignals (*white* arrows). *Abbreviations*: VGluT2, vesicular glutamate transporter 2; GABA, γ-aminobutyric acid; SOM, somatostatin.

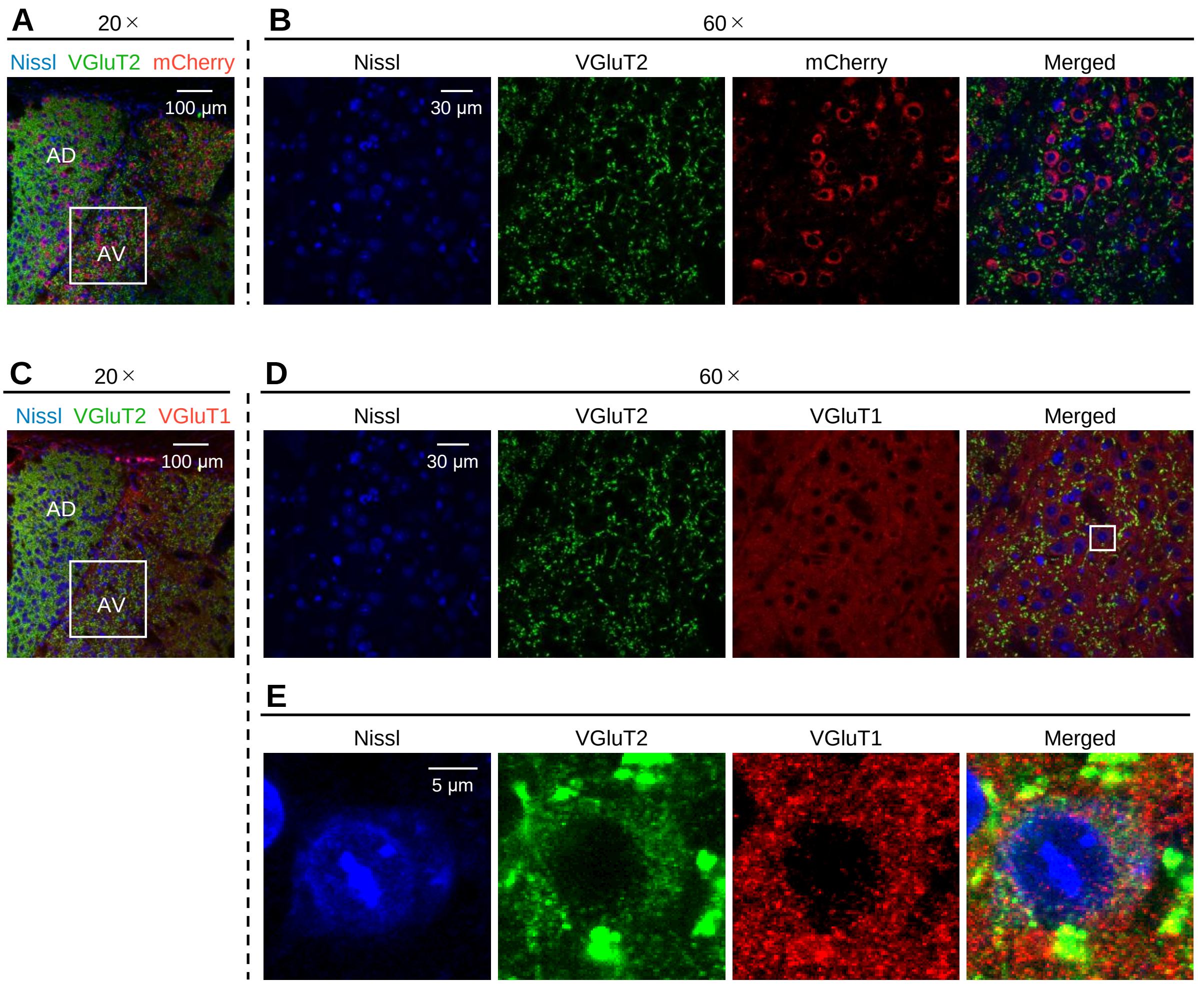

Supplementary Figure 11 | Expression of VGluT1 and VGluT2 in the thalamic injection site.

A, Representative image (20×) of the injection site. Nissl (*blue*), VGluT2 (*green*), and mCherry (*red*) signals are displayed. ***B***, High-magnification (60×) image of the boxed area in ***A***. Nissl (*blue*, *leftmost* (*first*)), VGluT2 (*green*, *second*), and mCherry (*red*, *third*) signals are presented. A merged image is displayed in the *fourth* panel. ***C***, The same as ***A***, but stained for Nissl (*blue*), VGluT2 (*green*), and VGluT1 (*red*). D, High-magnification (60×) image of the boxed area in ***C***. Nissl (*blue*, *leftmost* (*first*)), VGluT2 (*green*, *second*), and VGluT1 (*red*, *third*) signals are presented. A merged image is displayed in the *fourth* panel. ***E***, Enlarged images of the boxed area in ***D***. Nissl (*blue*, *leftmost* (*first*)), VGluT2 (*green*, *second*), and VGluT1 (*red*, *third*) signals are presented. A merged image is displayed in the *fourth* panel, showing that the neural soma subtly coexpresses VGluT1 and VGluT2; note that the intracellular VGluT1 and VGluT2 immunosignals are far less obvious than the extracellular VGluT2 immunosignals. *Abbreviations*: AD, anterior dorsal thalamus; AV, anterior ventral thalamus; VGluT1, vesicular glutamate transporter 1; VGluT2, vesicular glutamate transporter 2.

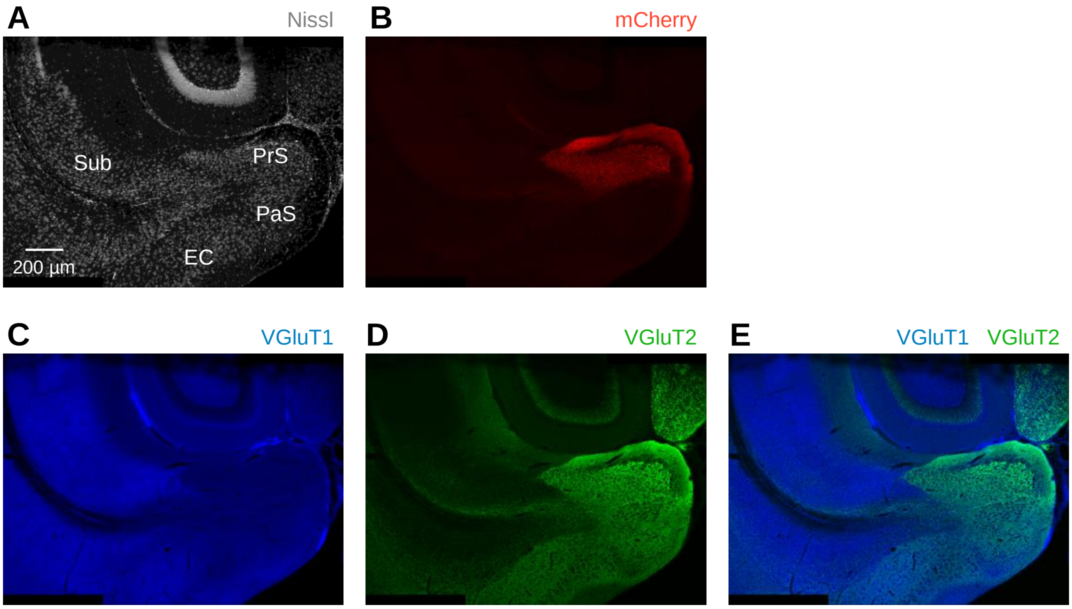

Supplementary Figure 12 | Few VGluT1 expression in the presubicular superficial layers. The brain slice was stained for Nissl substances, VGluT1, VGluT2, and simultaneously visualized with AAV-mediated anterograde tracing (mCherry). ***A***, Nissl signals (*gray*) in the parahippocampal region are displayed. ***B***, The same as ***A***, but for mCherry signals (*red*), which indicates anterograde tracing of the thalamic axonal projection. ***C***, The same as ***A***, but for VGluT1 immunosignals (*blue*). Note that the VGluT1 immunosignals are scarce in the presubicular superficial layers. ***D***, The same as ***A***, but for VGluT2 immunosignals (*green*) abundant in the presubicular superficial layers. ***E***, Merged image of VGluT1 (*blue*) and VGluT2 (*green*). These two proteins form a complementary expression pattern. *Abbreviations*: Sub, subiculum; PrS, presubiculum; PaS, parasubiculum; EC, entorhinal cortex; VGluT1, vesicular glutamate transporter 1; VGluT2, vesicular glutamate transporter 2.

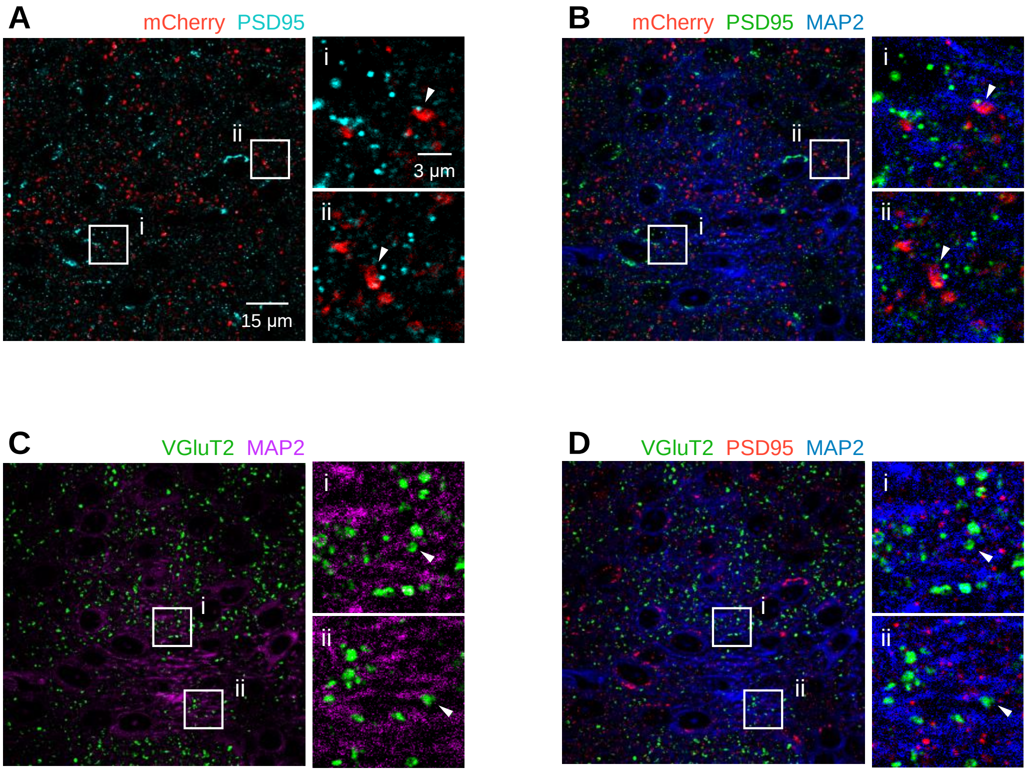

Supplementary Figure 13 | Thalamic axon terminals forming putative excitatory synapses impinging onto dendrites in the presubicular superficial layers.

Representative image (60× objective and 2× electronic zoom) of the presubicular superficial layers immunostained for VGluT2, PSD95, MAP2 and simultaneously visualized with AAV-mediated anterograde tracing. Putative excitatory synapses are indicated by *white* arrows. ***A***, *Left*: The signals of mCherry (*red*) and PSD95 (*cyan*) are presented. *Right*: Enlarged images of boxed areas **i** and **ii** (on the *left*) are displayed. Note that mCherry signals and PSD95 immunosignals are closely apposed to each other but do not overlap. ***B***, The same as ***A***, but for signals of mCherry (*red*), PSD95 (*green*), and MAP2 (*blue*). Putative synapses are confirmed based on the closely located signals of mCherry (presynaptic terminals) and PSD95 (postsynaptic densities). Note that most PSD95 signals were located close to MAP2 immunosignals indicative of neural dendrites and perikarya. ***C***, The same as ***A***, but for the immunosignals of VGluT2 (*green*) and MAP2 (*magenta*). Note that most VGluT2 and MAP2 immunosignals were closely located. ***D***, The same as ***A***, but for the signals of VGluT2 (*green*), PSD95 (*red*), and MAP2 (*blue*). *Abbreviations*: VGluT2, vesicular glutamate transporter 2; PSD95, postsynaptic density protein 95; MAP2, microtubule-associated protein 2.

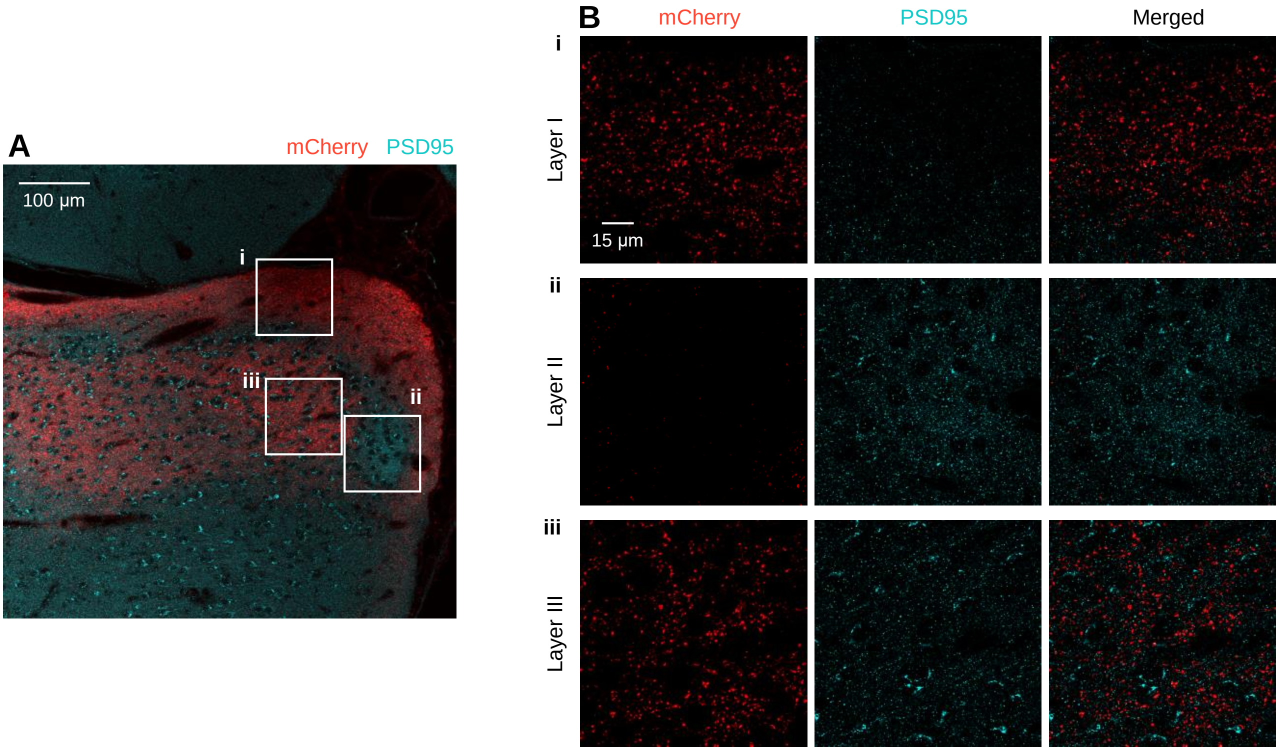

Supplementary Figure 14 | Distribution of putative synapses from thalamic axons onto neurons in the presubicular superficial layers.

A, Low-magnification (20× objective) image of the presubicular superficial layers. The slice was immunostained for PSD95 (*cyan*) and simultaneously visualized with AAV-mediated anterograde tracing (mCherry; *red*). ***B***, High-magnification (60× objective and 2 × electronic zoom) images of the boxed areas **i**, **ii**, and **iii** (in ***A***). mCherry signals (*red*, *leftmost* (*first*)) and PSD95 immunosignals (*cyan*, *second*) in layer I (**i**), layer II (**ii**), and layer III (**iii**) of the presubiculum are displayed. The merged images are displayed in the *third* column. *Abbreviation*: PSD95, postsynaptic density protein 95.

Supplementary Table 1 | Representative values of the spatial correlations.

	VGluT2 and mCherry	Nissl and VGluT2	Nissl and mCherry
600 μm	0.78 ± 0.08	0.34 ± 0.15	0.38 ± 0.16
900 μm	0.67 ± 0.08	0.17 ± 0.22	0.22 ± 0.18
1200 μm	0.71 ± 0.08	0.22 ± 0.21	0.23 ± 0.21
1500 μm	0.68 ± 0.11	0.22 ± 0.23	0.20 ± 0.25
1800 μm	0.69 ± 0.07	0.18 ± 0.22	0.16 ± 0.25
2100 μm	0.71 ± 0.07	0.24 ± 0.15	0.22 ± 0.20
2400 μm	0.72 ± 0.07	0.19 ± 0.21	0.20 ± 0.21
2700 μm	0.68 ± 0.08	0.13 ± 0.08	0.13 ± 0.11

(10× objective lens)

Data represent the mean ± SD (*n* = 5 or 6 mice (**Figure 3**)). *Abbreviation*: VGluT2, vesicular glutamate transporter 2.

Supplementary Table 2 | Statistics for spatial correlation.

	VGluT2 and mCherry *vs.* Nissl and VGluT2	VGluT2 and mCherry *vs.* Nissl and mCherry	Nissl and VGluT2 *vs.* Nissl and mCherry
600 μm	2.2×10^{-3}	4.4×10^{-3}	0.24
900 μm	2.6×10^{-3}	1.1×10^{-3}	0.74
1200 μm	5.0×10^{-3}	5.3×10^{-3}	>0.8
1500 μm	1.8×10^{-2}	2.0×10^{-2}	>0.8
1800 μm	1.7×10^{-3}	3.1×10^{-3}	>0.8
2100 μm	1.0×10^{-3}	4.5×10^{-3}	>0.8
2400 μm	4.7×10^{-3}	5.3×10^{-3}	>0.8
2700 μm	5.1×10^{-7}	1.6×10^{-4}	>0.8

Statistics are compiled by paired *t*-tests with *post hoc* Bonferroni correction (n = 5 or 6 mice (**Figure 3**)). *Abbreviation*: VGluT2, vesicular glutamate transporter 2.

Supplementary Table 3 | Statistics for the age-dependent cell density of interneurons.

	PV-expressing interneurons		SOM-expressing interneurons	
	JT	*P*	*JT*	*P*
600 μm	6.12	4.56×10^{-10}	1.27	0.10
900 μm	6.27	1.77×10^{-10}	-0.13	0.55
1200 μm	6.24	2.14×10^{-10}	0.34	0.37
1500 μm	5.86	2.38×10^{-9}	-0.19	0.58
1800 μm	6.32	1.32×10^{-10}	-0.67	0.75
2100 μm	4.73	1.14×10^{-6}	-1.40	0.92
2400 μm	3.93	4.26×10^{-5}	-1.98	†0.98
2700 μm	3.55	1.96×10^{-4}	-0.62	0.73

Statistics were collected by the Jonckheere-Terpstra test (one-tailed, n = 3 or 4 mice (**Figure 13**)). All P values are for increasing trends; †$P = 2.39 \times 10^{-2}$ for a decreasing trend. *Abbreviations*: PV, parvalbumin; SOM, somatostatin.
